# Decoding chemotherapy-induced peripheral neuropathy: ion channels, disease models, and future directions

**DOI:** 10.3389/fphar.2026.1762734

**Published:** 2026-06-23

**Authors:** Angela Lamberti, Eva Villalba-Riquelme, Asia Fernández-Carvajal, Antonio Ferrer-Montiel

**Affiliations:** 1 IDiBE - Instituto de Investigación, Desarrollo en Innovación en Biotecnología Sanitaria de Elche, Universidad Miguel Hernández, Elche, Alicante, Spain; 2 Molecular Nociception Group, Wolfson Institute for Biomedical Research, Division of Medicine, University College London, London, United Kingdom

**Keywords:** cancer, CIPN, ion channels, patients, preclinical models, sensory neurons, translational research

## Abstract

Chemotherapy-induced peripheral neuropathy (CIPN) is a significant side effect of cancer treatments like paclitaxel and oxaliplatin, impacting patient quality of life (QoL). Despite much research, its underlying mechanisms remain unclear and effective therapies are lacking. This review evaluates whether current experimental models accurately reflect CIPN, emphasizing recent molecular and cellular findings, especially the role of sensory ion channels. We outline limitations of existing models and propose more translational preclinical approaches to better capture disease complexity and patient variability. Improved models could enhance understanding of chemotherapy effects on peripheral nerves and support the development of interventions to prevent or mitigate CIPN. Prioritizing supportive care that minimizes treatment side effects may ultimately improve outcomes and patient wellbeing.

## Key points

- **CIPN is common and dose-limiting:** Up to 70% of patients develop CIPN during or shortly after neurotoxic chemotherapy, with 30% having persistent neuropathy at 6+ months.
- **Heterogeneous clinical presentation:** CIPN typically presents as a symmetric length-dependent sensory axonal neuropathy (stocking–glove distribution) with numbness, tingling, allodynia, and neuropathic pain. Motor deficits (distal weakness, gait imbalance) and autonomic features (e.g., orthostatic hypotension) occur in severe cases. However, it is still unclear why some patients develop stronger pain symptoms and others tolerate the treatment much better.
- **Mechanisms involve multi-organellar damage:** Diverse chemotherapeutics can injure peripheral neurons via microtubule disruption (axonal transport failure and distal degeneration), mitochondrial dysfunction and oxidative stress, neuroinflammation (activated immune and glial cells releasing cytokines), and ion channel remodeling.
- **Ion channel dysfunction is a key contributor:** Chemotherapy-induced alterations in ion channels (e.g., upregulation or hypersensitization of Nav, Ca^2+^ channels, HCN, and TRP channels, or downregulation of K_2_P channels or K_V_ channels) cause dorsal root ganglia neuron hyperexcitability. For example, platinum agents and taxanes may induce overactivity of TRPA1, TRPV1, TRPM8 and Na_V_1.7/1.8 channels, driving pain and dysesthesia.
- **No proven preventive treatments:** Notably, treatments commonly used for other neuropathies have limited effects against CIPN.
- **Emerging models and pipelines:** Advanced murine models (including transgenic mice, *in vivo* and *in vitro* optimized techniques, and refined pain-behavior assays) and human cellular models (DRG organotypic cultures, iPSC-derived sensory neurons) are enhancing mechanistic understanding but still face translational gaps. Computational modeling, along with AI of neuron excitability and drug–channel interactions now can complement experimental data. An integrative research pipeline that combines these approaches to address limitations of each technique is expected to identify novel therapeutic targets and improve predictive success in clinical trials.

## 1 Introduction

Chemotherapy-induced peripheral neuropathy (CIPN) is defined as an injury or degeneration of peripheral nerve fibers secondary to exposure to neurotoxic chemotherapy. CIPN typically manifests as a predominantly sensory neuropathy affecting the hands and feet in a glove-and-stocking distribution, often accompanied by dysesthesia (numbness, tingling), paresthesia, and neuropathic pain (Park et al., 2013). Motor symptoms (weakness, impaired fine motor skills) and autonomic symptoms (e.g., orthostasis, constipation) can co-occur, especially with certain drugs or in severe cases. These symptoms adversely impact patients Quality of life (QoL) and functional safety - for instance, CIPN patients have a significantly increased fall risk and disability in daily activities (Rahman et al., 2025). Although it is a long known unwanted and serious side-effect of most chemotherapeutics, supportive care strategies have not yet been implemented to prevent or reduce their impact on patients.

Clinically, CIPN is a dose-dependent toxicity. Neuropathic symptoms can develop during infusion or after the first chemotherapeutic cycle, usually becoming worse due to cumulative effects after multiple cycles. A landmark meta-analysis (Seretny et al., 2014) reported CIPN prevalence of ∼68% at 1 month, ∼60% at 3 months, and ∼30% at 6 months post-chemotherapy. Notably, up to 30% have chronic CIPN lasting ≥1 year. There are no approved agents to prevent CIPN (Hershman et al., 2014). Once CIPN developed, only *duloxetine* was recommended in the American Society of Clinical Oncology (ASCO) guidelines with limited efficacy.

The pathogenesis of CIPN is multifactorial and not yet fully elucidated. Chemotherapeutic agents have distinct primary mechanisms of action, e.g., microtubule disruption by taxanes/vinca alkaloids, DNA adduct formation by platinum agents, proteasome inhibition by bortezomib (Grisold et al., 2012). Nonetheless, they converge on common molecular pathways that produce peripheral neurotoxicity. Crucially, recent research highlights that many of these agents also induce ion channel dysregulation in sensory neurons. This includes altered expression or function of several families of ion channels. The existence of multiple targets notably complicates the development of single drug approaches, suggesting that targeting cellular crossroads or signaling integrators or using a phenotype-based strategy may be more clinically relevant and efficacious.

Animal models, especially rodents, have played a critical role in identifying the cellular and molecular mechanisms underlying CIPN (Naji-Esfahani et al., 2016; Eldridge et al., 2020). These models serve as the cornerstone of CIPN research and refining their clinical translation is vital for closing the gap between preclinical findings and their clinical application. A significant challenge remains the limited capacity to directly investigate these mechanisms in patients, as peripheral nervous tissue is accessible only from deceased donors or individuals undergoing a gangliectomy. Human cell-based platforms-such as induced pluripotent stem cells (iPSC)-derived neurons, organoids, and nerve-on-a-chip systems-are now offering complementary perspectives that more accurately represent human genetic and epigenetic factors, which are essential for advancing our understanding of CIPN pathophysiology, variability, and for testing therapeutic candidates (Mortensen et al., 2022; Han et al., 2024). Nevertheless, current preclinical models face notable limitations, particularly in their inability to assess the cognitive dimensions of CIPN, like the subjective intensity of numbness or tingling, which can only be evaluated in categorical terms.

This review examines CIPN using advanced methodologies for studying ion channels, in line with recent translational research efforts to enhance patient supportive care. It provides clinical context for CIPN and summarizes the neurotoxicity profiles of major chemotherapeutic classes. The role of ion channels in CIPN pathophysiology is explored by synthesizing evidence from both preclinical models and patient samples, while identifying areas where knowledge is lacking. The discussion includes an assessment of current preclinical CIPN models, such as human-based models (e.g., patient-derived sensory neurons), and new computational approaches that may improve the prediction of clinical outcomes. An integrated pipeline that combines preclinical and clinical findings to support the translation of mechanism-based therapeutic discoveries is proposed. The review concludes by outlining future directions and the potential clinical impact of multidisciplinary approaches for expanding supportive pharmacological options for cancer patients.

## 2 CIPN clinical context

CIPN symptoms can affect patients’ QoL and functional safety. For example, individuals with CIPN have an increased risk of falls and may experience difficulties in completing everyday activities. In addition to impact QoL, CIPN impacts cancer treatment protocols, and it is a leading cause of dose reductions and the discontinuation of chemotherapy (Mols et al., 2014; Battaglini et al., 2021). Major oncology guidelines recommend regular neurotoxicity monitoring and early intervention, typically by holding or reducing the chemotherapy dose if patients develop substantial neuropathy or functional impairment. The aim is to minimize irreversible nerve damage by modifying treatment when high-grade (≥ Grade 3) CIPN occurs. Nonetheless, dose adjustments intended to reduce CIPN risk may affect oncologic effectiveness if made prematurely, presenting a challenge for clinicians seeking to balance therapeutic benefits with neurologic risk. In clinical settings, a notable proportion of patients receive less chemotherapy than initially planned due to neuropathy. For instance, in an adjuvant colon cancer trial, approximately 20%–30% of patients receiving oxaliplatin discontinued the drug early because of CIPN (Żok et al., 2021). Many cancer survivors continue to experience chronic CIPN symptoms requiring ongoing management from primary care physicians or neurologists.

CIPN poses significant management challenges. There are no approved agents to prevent CIPN - all major randomized trials of prophylactic therapies have been negative or inconclusive (Loprinzi et al., 2020; Derman and Davis, 2021). Once CIPN develops, treatment options are limited. Standard analgesics (opioids, NSAIDs) have minimal effect on neuropathic symptoms like tingling or numbness. To date, duloxetine (a serotonin–norepinephrine reuptake inhibitor) is the only therapy with evidence supporting its use in the treatment of CIPN (Wang et al., 2022). Beyond duloxetine, evidence is weak or conflicting for other pharmacologic treatments and for non-pharmacologic interventions, although small studies suggest potential benefit in mitigating symptoms or improving functional capacity, as discussed in Section 7.6. Thus, CIPN management often relies on dose modification of the offending chemotherapy and multidisciplinary supportive care.

In summary, CIPN is a pervasive complication of oncology care, with profound implications for patient wellbeing and treatment outcomes. This pressing clinical problem motivates efforts to better understand its mechanisms and to develop effective preventative strategies.

## 3 Chemotherapeutic agents and neurotoxicity

### 3.1 Incidence and clinical features

The incidence and severity of CIPN vary widely among chemotherapeutic drugs. The highest risk is associated with platinum-based drugs (such as oxaliplatin) and taxanes (such as paclitaxel). Oxaliplatin causes acute cold-triggered paresthesia in nearly all patients and chronic peripheral neuropathy in ∼70%, while paclitaxel causes CIPN in 60%–80% of patients, with 25%–30% experiencing moderate to severe CIPN (Grade ≥2) (Saif and Reardon, 2005; Kang et al., 2021). By contrast, carboplatin, a less potent platinum-based drug, exhibits lower neurotoxicity (4%–30% incidence) (McWhinney et al., 2009). Among newer or less common neurotoxic agents, the epothilone ixabepilone resembles taxanes in producing significant sensory neuropathy, whereas targeted therapies like immune checkpoint inhibitors rarely cause peripheral neuropathy, although they often cause pruritus (Salinas et al., 2021), another sensory dysfunction.

CIPN risk generally escalates with cumulative dose exposure. There are often threshold doses beyond which neuropathy incidence rises steeply. For instance, cisplatin-induced neuropathy tends to emerge around 300 mg/m^2^ total dose, with ∼90% of patients affected by the time 500–600 mg/m^2^ is reached (Alberts and Noel, 1995). Paclitaxel neuropathy usually remains mild in early cycles but increases markedly once cumulative doses exceed ∼1,000–1,400 mg/m^2^ (Starobova and Vetter, 2017). Recognizing these dose-dependent patterns is critical for clinicians to monitor symptoms and adjust dosing schedules proactively. Some protocols use “stop-and-go” strategies (pausing a neurotoxic drug after a set number of cycles and resuming later if neuropathy improves) to mitigate cumulative nerve damage, particularly for oxaliplatin in colorectal cancer, though this approach is still being refined in clinical trials. Risk factors for developing severe or persistent CIPN include pre-existing neuropathy (e.g., diabetes), advanced age, cumulative dose and dose intensity of neurotoxic agents, and certain genetic predispositions (Kerckhove et al., 2017).

### 3.2 Mechanisms of neurotoxicity by chemotherapeutic agent class

Different chemotherapeutic drugs have distinct primary mechanisms of action against tumors, and these translate to differing neuropathy phenotypes and injury mechanisms in the peripheral sensory system. Platinum compounds (such as cisplatin and oxaliplatin) form DNA crosslinks/adducts in tumor cells halting proliferation (Bernges and Holler, 1991). As an off-target effect these drugs damage DNA in dorsal root ganglia (DRG) neurons promoting a sensory neuronopathy–injury to the DRG cell bodies–with a “dying back” axonal degeneration of distal sensory fibers (Cashman and Höke, 2015). Oxaliplatin also chelates calcium and triggers acute axonal hyperexcitability (hence the acute cold neuropathy), whereas cisplatin accumulates in DRG neurons and causes cumulative neuronal loss (Acklin and Xia, 2021; Kang et al., 2021).

Taxanes (paclitaxel, docetaxel) and vinca alkaloids (vincristine, vinblastine) both target microtubules but in opposite ways. Whereas taxanes stabilize microtubule polymers, vinca alkaloids depolymerize microtubules (Park et al., 2013; Hammad et al., 2023). Both mechanisms disrupt axonal transport, leading to axonal swelling and degeneration, especially in the longest peripheral nerves (length-dependent axonopathy). Clinically, taxane neuropathy is largely sensory. Paclitaxel can also produce an acute syndrome of diffuse aches and neuropathic pain (paclitaxel acute pain syndrome) that peaks a few days after infusion, which is thought to result from acute nerve inflammation and is predictive of later neuropathy severity (Loprinzi et al., 2007). Vinca alkaloids, especially vincristine, often cause a mixed sensorimotor neuropathy (Li et al., 2024b). In addition to distal numbness and paresthesia, vincristine commonly induces significant motor weakness (e.g., foot drop, hand weakness) and autonomic dysfunction (constipation, orthostatic hypotension) (Li et al., 2024b). Almost all patients receiving standard vincristine dose will develop some neuropathy, particularly for cumulative doses beyond ∼4 mg/m^2^, forcing dose-capping to limit neurotoxicity (Grisold et al., 2012). Notably, pharmacogenomic factors influence vincristine neuropathy. For instance, children with acute lymphoblastic leukemia who carry a variant in the tubulin gene CEP72 have shown higher neuropathy incidence, suggesting a future role for genotype-guided dosing (Uittenboogaard et al., 2022).

Proteasome inhibitors (most prominently bortezomib, used in multiple myeloma) cause a distinctive CIPN that is typically a painful, small-fiber sensory neuropathy. Bortezomib-induced peripheral neuropathy often presents with burning dysesthesia in the feet and can be dose-limiting in about 5%–15% of patients on the IV formulation (Yamamoto and Egashira, 2021). Mechanistically, bortezomib proteasome inhibition leads to accumulation of misfolded or ubiquitinated proteins in neurons, triggering an endoplasmic reticulum stress response and mitochondrial dysfunction in peripheral nerve fibers (Sogbein et al., 2024). Bortezomib particularly affects sensory neurons in the DRG and small nerve fibers; pathological studies show loss of intraepidermal nerve fibers (IENF) in the skin of treated patients (Bechakra et al., 2018), corresponding to the clinical small-fiber neuropathy (Han and Smith, 2013). Bortezomib neurotoxicity has been mitigated in practice by changing the dosing schedule and route. For instance, weekly and subcutaneous bortezomib results in lower peak drug levels than IV bolus, roughly halving the incidence of neuropathy (Moreau et al., 2011). Nonetheless, when severe neuropathy or pain develops on bortezomib treatment the dose is reduced or patient switched to an alternative drug such as the second-generation proteasome inhibitor carfilzomib, which has much lower neurotoxic risk (Siegel et al., 2013).

Despite their diverse primary targets, these neurotoxic agents converge on common pathophysiological pathways in the peripheral nervous system. These events disrupt the homeostasis of peripheral neurons and their supporting glia, culminating in an axonopathy from the distal terminals. Shared features of CIPN across drug classes include distal axonal degeneration, mitochondrial damage with resultant oxidative stress, de-myelination, and activation of the neuroimmune system (e.g., recruitment of macrophages and glial cells that release proinflammatory cytokines) (Zajaczkowską et al., 2019). This leads to a final common pathway of nerve fiber loss and dysfunctional nerve signaling. Moreover, it has become evident that ion channel dysregulation is a unifying element in CIPN caused by many agents (Park et al., 2008; Nassini et al., 2013). Chemotherapy often induces maladaptive changes in the expression or function of neuronal ion channels, which contributes substantially to neuropathic symptoms (Cabañero et al., 2022).

## 4 Pre-clinical CIPN models

**FIGURE 1:**
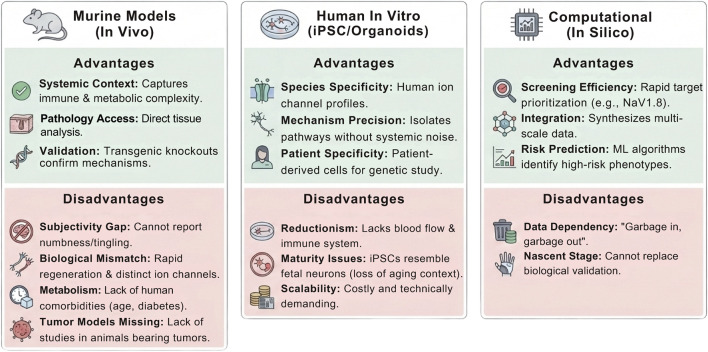
Comparative overview of the main advantages and limitations of the three major preclinical approaches used in chemotherapy-induced peripheral neuropathy (CIPN) research: murine *in vivo* models, human *in vitro* platforms (including iPSC-derived systems and organoids), and computational *in silico* approaches.

Preclinical disease models are instrumental to advance our knowledge on the underlying molecular mechanism giving rise to the illness and to screen for drug candidates that may be developed into useful drugs for treating the investigated condition. Currently, two major types of preclinical models are used to investigate the molecular correlations of CIPN. We describe these models next, and their main advantages and disadvantages are summarized in Figure 1.

### 4.1 Murine models

Animal models, especially murine (mouse and rat) models, have been indispensable for studying CIPN and developing potential interventions. By replicating key features of human CIPN in rodents, researchers have been able to uncover its underlying mechanisms in a controlled environment and to test experimental treatments aimed to reduce the neuropathy or prevent its chronification.

Murine models effectively replicate numerous clinical manifestations of CIPN. For instance, mice administered paclitaxel exhibit quantifiable allodynia and hyperalgesia, which parallel the tactile, heat and cold hypersensitivity observed in patients (Materazzi et al., 2012). Rodents treated with oxaliplatin display cold-provoked behaviors—such as rapid paw withdrawal on a cold plate—and gait disturbances comparable to the cold intolerance and balance difficulties experienced by individuals with CIPN (Nassini et al., 2013). These phenotypes facilitate objective assessment of pain sensitivity using assays such as von Frey filaments for mechanical thresholds and acetone drop or cold plate tests for evaluating cold sensitivity. Additionally, murine models permit direct pathological examination of nervous tissue, an approach not feasible in living patients. Recent advances in *vivo* imaging techniques further enable real-time monitoring of sensory neuron responses within the physiological context of a living organism (Iseppon et al., 2023; MacDonald et al., 2021).

Researchers have studied DRG, peripheral endings, and skin from rodents with CIPN, finding changes such as distal axon degeneration, loss of intraepidermal nerve fibers, and mitochondrial abnormalities in affected nerves (Park et al., 2023). Rodent studies have reported alterations in ion channel expression in DRG neurons, suggesting chemotherapy-induced channelopathies. Transgenic mouse models are used to investigate mechanisms; for example, knocking out specific genes can clarify their roles in CIPN. For instance, TRPA1 knockout mice show resistance to oxaliplatin-induced cold hyperalgesia, indicating involvement of TRPA1 (Zhao et al., 2012). Likewise, studies in rats have shown that blocking toll-like receptor (TLR)-4 or its adaptor MyD88 reduces paclitaxel-induced mechanical hypersensitivity, implicating innate immune signaling in CIPN pathogenesis (Li et al., 2014). Murine models are also employed as preclinical platforms for evaluating potential therapies. Various agents have been tested in these rodent models before human trials, including duloxetine, which was shown in rats to reduce bortezomib- and paclitaxel-induced hypersensitivity and later advanced to clinical trials (Mansooralavi et al., 2023). Compounds that demonstrate no effect in animal models may be deprioritized, while those showing protective or anti-neuropathic effects (e.g., some antioxidants, cytokine inhibitors, or ion channel blockers) may be selected for clinical testing. Overall, animal models have contributed to our understanding of CIPN pathogenesis - including microtubule transport disruption, oxidative stress, inflammation, and ion channel dysfunction - and are used for hypothesis development and preliminary evaluation of interventions (Flatters et al., 2017; Lv et al., 2023).

Although murine models offer certain advantages, they present notable limitations when modeling CIPN as experienced by patients. Rodents are unable to convey subjective symptoms such as numbness, tingling, or spontaneous pain; thus, researchers must rely on surrogate behavioral measures to infer these experiences. Standard assays - including paw withdrawal thresholds and thermal response tests - primarily assess evoked pain reflexes, potentially underrepresenting sensory loss or ongoing dysesthesias. A mouse may exhibit normal motor function and lack abnormal withdrawal responses, yet the possibility of unreported “numb” sensations remains, as these deficits could go undetected in animal studies (Jolivalt et al., 2016). Furthermore, some behaviors observed in rodents, such as excessive grooming or limb shaking, may not correspond directly to human clinical manifestations. Hence, the modelling of predominantly non-evoked symptoms, such as persistent numbness and tingling, which are both commonly reported by CIPN patients, remains a challenge.

An additional limitation is the more rapid recovery from neuropathy observed in rodents compared to humans (Flatters et al., 2017). In many murine models, CIPN symptoms peak and subsequently partially resolve within weeks following cessation of chemotherapy, whereas in clinical populations, neuropathy may persist for months, years, or even become irreversible. Young mice also possess robust nerve regeneration capabilities, including regrowth of intraepidermal nerve fibers over several weeks, while adult humans experience markedly slower, if any, nerve regeneration (Griffin et al., 2010). Consequently, treatments that appear effective in resolving CIPN in mice may simply benefit from spontaneous recovery phenomena not replicated in humans (Park et al., 2013). Differences in drug dosing and metabolism further complicate translational efforts, as murine protocols frequently use condensed dosing schedules designed to induce neuropathy within the species’ short lifespan, which may not accurately reflect human chemotherapy regimens.

Mice also metabolize drugs at a quicker rate, and they generally lack comorbidities, such as diabetes or concurrent medication use, which are present in human patients. These differences may cause divergences in experimental outcomes when comparing animal models and humans (Flatters et al., 2017). There are also species-specific variations in nerve biology; for example, certain ion channel subtypes in DRG neurons show different expression patterns in mice compared to humans, and rodents often demonstrate more significant remyelination and axonal regeneration than humans (Cayre et al., 2021; Qi et al., 2024). As a result, pathological mechanisms that are predominant in mice may not be as relevant in humans, and vice versa. These factors contribute to the challenges in translating therapies effective in rodent CIPN models to successful clinical trial results.

A commonly overlooked limitation is the homogeneous genetic background of mouse and rat strains (Gurwitz et al., 2001). While this uniformity facilitates reduced data variability and helps achieve statistical significance, it does not reflect the genetic diversity seen in humans affected by CIPN. Including more rodent strains in preclinical studies could address this issue to some extent, but would also increase animal usage, which conflicts with the principles of the 3 R rule. Furthermore, expanding strain testing does not necessarily improve the clinical relevance of the findings, as testing would still occur in genetically similar animals.

Studies investigating the effects of chemotherapeutic drugs on animal models with cancer remain limited, likely due to the increased complexity, the presence of opposing mechanisms (e.g., tumor reduction leading to decreased pain symptoms), and ethical restrictions. However, such models could better represent the pathophysiology experienced by oncology patients, as tumors and the organism’s responses can also sensitize sensory neurons (Haroun et al., 2022). For example, Kim H. K. et al. (2025), reported earlier onset and greater severity of paclitaxel-induced pain behaviors in breast tumor-bearing mice.

Limitations related to animal procedure approvals and increasing costs further constrain the study of chronic CIPN using animal models (Thulin et al., 2014). Although clinical and *in vivo* data remain essential, information from organ-on-a-chip models, cell lines, and previous research can support the drug discovery process by enabling earlier identification of toxicities and potentially reducing time and resource expenditure.

### 4.2 Human *in vitro* models

Advancements in murine *in vitro* models that effectively replicate the key features of CIPN observed *in vivo* have paved the way for the utilization of human cellular models (Table 1; Riva et al., 2018; Villalba-Riquelme et al., 2022). Cultured human sensory neurons, including those derived from iPSCs, facilitate detailed analysis of mechanism-specific pathways and enable the evaluation of targeted interventions (Mortensen et al., 2022). Notably, these human neuronal models capture unique elements - such as human-specific ion channel profiles - that may be absent in animal studies, providing an opportunity to test therapies directly on human neural tissue. The primary sources for these cultures include donor organs, iPSC-derived sensory neurons, and transdifferentiated fibroblasts.

**TABLE 1:** Recent human and murine *in vitro* and *in silico* CIPN models and their key features.

Murine *in vitro* models		
Chemotherapeutic	CIPN features	References
Bortezomib	↑Delta 2 tubulin accumulation ↑Axonal injury	Pero et al. (2021)
Oxaliplatin	Increased IB4(+) nociceptors excitability ↑ TRPV1 ↑TRPA1 ↑Na_V_1.8 mediated currents Female nociceptors showing increased excitability	Villalba-Riquelme et al. (2025)
Paclitaxel	Mouse DRG in compartmentalized primary cultures ↑ TRPV1 ↑ TRPA1 calcium signals and ↑ response to KCl	Giorgi et al. (2023)
Paclitaxel	Increased IB4(−) and IB4(+) nociceptors excitability ↑ TRPV1 ↑TRPM8 ↑Na_V_1.8 mediated currents Female nociceptors showing increased excitability	Villalba-Riquelme et al. (2022)
Paclitaxel	Perturbed integrins trafficking	Shin et al. (2021)
Paclitaxel	↑ spontaneous activity ↑stress and inflammatory markers ↑pERK ↑pp38 ↑IL6 ↑CCL2	Li et al. (2021)
Bortezomib, Oxaliplatin Paclitaxel, or Vincristine	3D in vitro model exposed to bortezomib, oxaliplatin, paclitaxel, or vincristine individually Altered nerve conduction velocity and amplitude	Kramer et al. (2020)

Human *in vitro* models		
hiPSC-derived sensory neurons		
Chemotherapeutic drug	CIPN features	References
Bortezomib, Cisplatin Doxorubicin 5-fluorouracil, Paclitaxel, or Vincristine	For paclitaxel, vincristine, bortezomib, and cisplatin: axonal blebbing and lower cell viability For paclitaxel: ↑neuronal injury markers ATF3, SESN2, CASP3, JUN, BBC3, and HRK; ↑ vasoactive substances and pain inducing neuropeptides such as CALCB and ADM; and ↑pain associated gene PIRT; and inflammation associated genes such as TNFRSF9, TNFRSF10B, or MMP10	Schinke et al. (2021)
Oxaliplatin	Mechanical allodynia ↑TRPV1 stimulation response	Holzer et al. (2022)
Paclitaxel	hiPSCs from CIPN patients Uncharacterized	Lewis et al. (2025)
Paclitaxel	Axonal retraction and reduced neuronal network Increased TRPV1 mRNA levels	Mortensen et al. (2024)
Vincristine	Reduced neurite length	Petrova et al. (2024)
Vincristine	Axonal fragmentation Increased TRPV1 mRNA levels	Mortensen et al. (2024)
Organoids		
Vincristine	3D-model - deficits in nerve conduction velocity, and axonal degeneration	Rountree et al. (2025)

*In silico* models		
Chemotherapeutic drug	CIPN features	References
Paclitaxel-like phenotype	↑Na_V_1.8 ↓K_DR_ as crucial determinants of nociceptor’s spontaneous activity induced by paclitaxel	Verma et al. (2020)

Donor-derived DRG retain the heterogeneity of peripheral nerves, making them a highly translational model for mechanistic investigations involving diverse neuronal populations. Studies utilizing this approach have reported increased TRPV1 and Na_V_1.7 activity following paclitaxel administration (Li et al., 2015; 2018). However, availability of human DRG tissue is highly limited due to its reliance on cadaveric donors, restricting the scalability of this resource. Consequently, there is growing emphasis on utilizing iPSCs to generate sensory neurons for research purposes (Table 1).

iPSC-derived sensory neurons (iPSC-SN) have been demonstrated to replicate certain morphological alterations reported in chemotherapy-treated patients and animal models, such as the axonal retraction observed after treatment with taxanes or vinca-alkaloids (Table 1) (Mortensen et al., 2024; Petrova et al., 2024). Additionally, iPSC-SN models demonstrate increases in the expression of neuronal injury markers, TRPV1 mRNA levels, TRPV1-induced responses, and in the levels of other pain-related markers (e.g., CGRP), providing support for the use of these models for CIPN research (Table 1) (Schinke et al., 2021; Holzer et al., 2022; Mortensen et al., 2024).

iPSC-derived sensory neurons have also been generated from CIPN patients, providing a model in which cells are sensitized within their original genetic context (Lewis et al., 2025). This approach preserves the patients’ genetic backgrounds. However, iPSC-SN human cultures tend to show limited differentiation efficiency and reproducibility, and they do not fully recapitulate the functional diversity of native sensory neurons; moreover, cellular reprogramming can reset age-related and epigenetic features, meaning patient-derived iPSC neurons may not preserve the donor’s original epigenetic imprinting (Mertens et al., 2015; Kalia et al., 2024).

Because multiple cell types contribute to CIPN, more complex *in vitro* models may better represent its pathology. Traditional cell cultures lack the nervous system and immune complexity of living organisms, but organoids and 3D cultures offer improved neuronal microenvironments. Emerging 3D organotypic cultures, like human DRG organoids and skin-nerve explants, are now being used to model CIPN. Rountree et al. (2025) found that a 3D co-culture of sensory neurons and Schwann cells showed nerve conduction deficits and axonal degeneration, effectively replicating neuropathic changes (Rountree et al., 2025). Although still developing and technically demanding, these advanced models could prove more translatable than standard iPSC 2D cultures.

To scale the proposed human iPSC-SN and 3D organoid/organotypic platforms into drug-discovery-grade assays, it is important to integrate them with high-content screening (HCS) workflows that combine automated microscopy with robust, multi-parametric image analytics so that disease-relevant phenotypes can be quantified at scale rather than inferred from single endpoints. In 2D iPSC-neuron formats, axonal integrity can be captured by immunolabelling neuronal processes (e.g., βIII-tubulin) and quantified as complementary morphometric features (e.g., total neurite/axon area or length and network complexity/branching) using established high-content analysis (HCA) pipelines for iPSC-derived neurons (Lickfett et al., 2022) coupled to open-source analysis software such as CellProfiler (Carpenter et al., 2006). Critically, in a human iPSC-SN (iPSC-SN) CIPN model, paclitaxel produces dose- and time-dependent loss of neurite network complexity detected by βIII-tubulin staining and high-content imaging, supporting neurite/network metrics as scalable, pharmacodynamically sensitive readouts of axonopathy (Xiong et al., 2021). In parallel, mitochondrial health can be profiled in the same multiwell format by incorporating live-cell imaging readouts of mitochondrial membrane potential (Zink et al., 2022) and, where higher dimensionality is needed, broader multi-parametric mitochondrial feature sets, including mitochondrial content, fragmentation state, and mitochondrial ROS (Little et al., 2020). Notably, paclitaxel also decreases mitochondrial membrane potential and slows mitochondrial movement along neurites in iPSC-SNs, providing a strong rationale for combining axon- and mitochondria-related endpoints in the same HCS assay to align readouts with proposed mechanisms (Xiong et al., 2021). For 3D organoids, high-content drug screening is increasingly supported by automation-compatible, multiwell workflows using confocal imaging for image-based phenotyping (Bozal et al., 2024), consistent with broader efforts to operationalize organoids for high-throughput/high-content screening (Lampart et al., 2023). Finally, as feature dimensionality becomes a practical bottleneck, machine-learning approaches can improve sensitivity and the throughput of phenotypic classification, as demonstrated by morphological deep-learning pipelines applied to human iPSC-derived neurons for peripheral neurotoxicity prediction (Han et al., 2024).

## 5 Toward a more translational preclinical approach: an integrated pipeline

**FIGURE 2:**
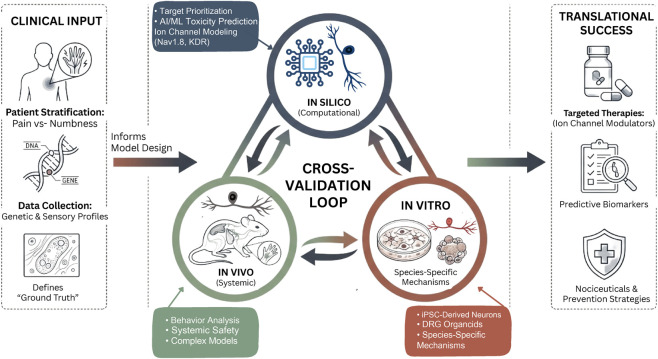
Integrated translational pipeline for CIPN research. Schematic overview of an integrated translational pipeline for chemotherapy-induced peripheral neuropathy (CIPN) research. AI/ML, artificial intelligence/machine learning; iPSC, induced pluripotent stem cell; DRG, dorsal root ganglion; Na_v_1.8, voltage-gated sodium channel subtype 1.8; **K**_**DR**_, delayed rectifier potassium channel.

An effective integrated pipeline for CIPN research unites *in vivo* animal models, human *in vitro* systems, and *in silico* computational tools to systematically cross-validate data and bridge the translational gap between laboratory discoveries and patient care (Figure 2). Within this multidisciplinary framework, murine models remain the indispensable foundational step for uncovering complex neurobiological mechanisms, evaluating whole-organism behavioral phenotypes (such as cold and mechanical hypersensitivity), and conducting preliminary safety testing. However, because rodents lack human genetic diversity and cannot communicate subjective sensory deficits like numbness, the pipeline necessitates integrating these *in vivo* findings with advanced human *in vitro* platforms. Utilizing human iPSC-SNs, patient-donor tissue, and 3D organotypic cultures allows researchers to validate therapeutic targets - such as human-specific ion channel profiles - within a biologically accurate human genetic and epigenetic context. Concurrently, computational modeling and AI act as the predictive and analytical engine of the pipeline. These *in silico* approaches integrate multi-scale experimental data to screen and prioritize relevant protein targets, simulate drug-ion channel interactions, and predict neurotoxicity profiles before extensive physical testing occurs. Finally, this pipeline is continuously refined by integrating real-world clinical findings, such as biomarker identification and patient imaging. By iteratively combining target identification in animals, validation in human cells, and AI-driven predictive modeling, this integrated approach comprehensively captures the complexity of CIPN and is expected to significantly improve the predictive success of novel interventions in clinical trials.

## 6 Ion channels involved in CIPN pathophysiology

**FIGURE 3:**
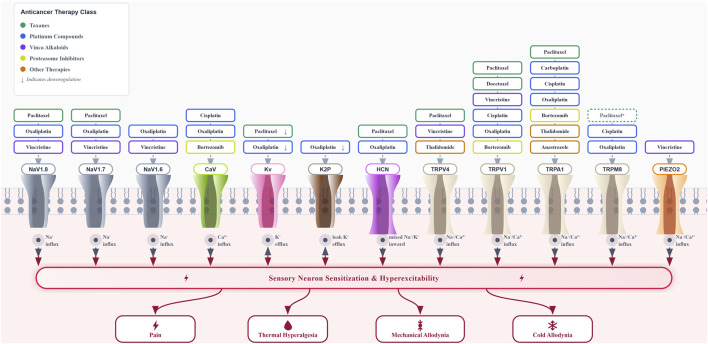
Ion channels involved in CIPN pathophysiology and their associations with anticancer therapies. Schematic representation of the main ion channels implicated in chemotherapy-induced peripheral neuropathy, their modulation by different classes of anticancer agents, and their contribution to sensory neuron sensitization, hyperexcitability, and CIPN-related symptoms. Dashed outlines indicate controversial or suggested associations. Differential ion channel involvement likely contributes to distinct CIPN phenotypes: cold allodynia, mainly linked to TRPM8 and TRPA1, while Na_**V**_1.6/1.7/1.8 might amplify cold-evoked firing and K2P downregulation could enhance cold sensitivity; mechanical allodynia/hypersensitivity, associated with PIEZO2, TRPV4, TRPA1, Ca_**V**_, and Na_**V**_1.6/1.7/1.8; thermal hyperalgesia, primarily mediated by TRPV1, although TRPA1 and TRPV4 might contribute as well and Na_**V**_ **1.7/**1.8 might amplify this sensation; and spontaneous pain/paresthesia, mainly driven by Na_**V**_1.6/1.7/1.8, HCN, Ca_**V**_, and loss of repolarizing Kv and K2P channel activity.

Multiple lines of evidence from preclinical models indicate that CIPN is not due solely to structural nerve damage but also involves profound changes in the electrical excitability of peripheral neurons. In CIPN, there is often an upregulation of excitatory ion channels and/or downregulation of inhibitory channels on sensory neurons. These channelopathies heighten neuronal activity, leading to abnormal pain signaling (neuropathic pain) and paresthesia. Key channel types implicated in CIPN include voltage-gated sodium (Na_v_), potassium (K_v_) and calcium (Ca_v_) channels, the two-pore domain potassium channels (K_2_P), hyperpolarization-activated cyclic nucleotide-gated cation channels (HCN), and the transient receptor potential (TRP) family of cation channels. These ion channel alterations and their reported associations with different chemotherapeutic agents are summarized in Figure 3.

### 6.1 Voltage-gated sodium channels (NaV)

Voltage-gated sodium channels are critical for action potential generation in nerve fibers. CIPN models show that certain Na_V_ subtypes become abnormally upregulated or hyperactive after chemotherapy. For instance, paclitaxel treatment increases the expression of Na_V_1.7 channels in dorsal root ganglion neurons, which appears to increase subthreshold membrane potential fluctuations and lowers the voltage threshold for action potential firing promoting spontaneous ectopic discharges (a source of neuropathic pain). An increase in Na_V_1.8-mediated currents has also been observed (Villalba-Riquelme et al., 2022), and computational modeling predicts that paclitaxel-induced hyperexcitability is highly sensitive to Na_V_1.8 activity. Consistently, mice lacking Na_V_1.8 or those treated with Na_V_-blocking drugs show reduced pain behaviors in some CIPN models (de Lima et al., 2024). Notably, individual genetic differences in sodium channels may partly explain variability in CIPN severity: a polymorphism in SCN10 A (the gene encoding Na_V_1.8) was linked to oxaliplatin neuropathy severity in one study, suggesting that patients with certain channel genotypes could be more susceptible to CIPN (Argyriou et al., 2006). These findings highlight Na_v_ channels as drivers of both nociceptor excitability and of CIPN sensory symptoms, validating their status as interesting therapeutic targets. In an adult mouse nociceptor culture model of CIPN (involving repeated-paclitaxel doses applied to primary sensory neurons), we previously demonstrated that cumulative paclitaxel exposure produced persistent spontaneous and evoked hyperexcitability, an increase in Na_V_ current, and a significant rise in Na_V_1.8 mRNA after the second exposure (Lamberti et al., 2026). These results provide further evidence that NaV1.8 is a key contributor to paclitaxel-induced excitability. However, clinical trials with mexiletine, a sodium channel blocker, have reported only modest symptom improvements in CIPN patients. Another potential candidate for the treatment of CIPN is the recently approved Nav1.8 blocker suzetrigine (Jones et al., 2023). Although suzetrigine is not currently being clinically tested for CIPN, it is under investigation for other neuropathic conditions, such as diabetic neuropathy Vertex Pharmaceuticals Incorporated (2026).

### 6.2 Voltage-gated potassium channels (K_V_)

K_V_ channels normally act as a brake on neuronal excitability by repolarizing the membrane and limiting action potential firing. Chemotherapy can cause a loss of this inhibitory control. Paclitaxel-exposed neurons show a reduction in K_V_ currents (partly due to downregulation of specific K_V_ channel subunits), which prolongs depolarizations and enhances excitability (Villalba-Riquelme et al., 2022). In silico neuron models identified the decrease in K_V_ conductance as a key contributor to paclitaxel-induced hyperexcitability, synergizing with Na_V_ upregulation (Verma et al., 2020). Experimentally, enhancing K_V_ channel activity can counteract this effect. Indeed, applying a K_V_7.2 channel opener (retigabine) or exogenous PIP_2_ (which boosts certain K_V_ currents) normalized the firing rate in paclitaxel-treated neurons (Li et al., 2019; Wu et al., 2022). These observations suggest that strategies to increase potassium currents or stabilize neuronal resting potential might reduce CIPN symptoms. However, specific K_V_ -targeted treatments for CIPN have not yet reached the clinic.

### 6.3 Voltage-gated calcium channels (Ca_V_)

Voltage-gated calcium channels (Ca_v_), particularly N-type Ca_V_2.2 and T-type Cav3.2 subtypes, play roles in neurotransmitter release and pain pathways. Although they are less extensively studied in CIPN, there is cumulative evidence of their involvement in acute and chronic pain. Oxaliplatin, for example, can alter calcium signaling in sensory neurons, possibly by affecting Ca_V_ channel function or expression (Schmitt et al., 2018). The observed disturbances in calcium signaling may interfere with nerve excitability, neurotransmission,or the release of pro-inflammatory peptides. Consistent with the role of calcium influx in CIPN pain, intrathecal ziconotide, a selective N-type Ca_V_ blocker, is an effective analgesic for refractory neuropathic pain (McDowell and Pope, 2016). It is used in severe cases of pain generally but has not been specifically approved for CIPN. More research is needed to clarify how chemotherapy affects Ca_V_ activity and whether channel modulators could ameliorate CIPN symptoms without causing systemic side effects.

### 6.4 K_2_P channels

K_2_P ion channels are major determinants of the background K^+^ currents that establish the resting membrane potential. Downregulation of TREK-1 and TRAAK mRNA has been identified in a mouse model treated with oxaliplatin (Descoeur et al., 2011). In addition, oxaliplatin appears to modulate the activity of TREK channels by a pH-dependent mechanism (Dionisi et al., 2020).

### 6.5 HCN channels

HCN ion channels are activated by hyperpolarization and the presence of cyclic nucleotides, allowing the flux of K^+^ and Na^+^. These channels are activated in the after-hyperpolarization phase (AHP) of the action potential and play a fundamental role restoring the resting membrane potential. Alteration of their expression and function can elicita faster recovery from the AHP or a significant attenuation of the phase, leading to an increase in the firing frequency, including spontaneous activity. Notably, HCN1 mRNA upregulation has been described in mice treated with paclitaxel or oxaliplatin (Descoeur et al., 2011; Zhang and Dougherty, 2014), and may contribute to the neuropathy provoked by both chemotherapeutic drugs.

### 6.6 TRP channels

The transient receptor potential (TRP) channels constitute a family of non-selective cation channels, many of which function as sensory transducers that detect thermal, mechanical, or chemical stimuli. Thermosensory TRP channels expressed in peripheral nociceptive endings play a critical role in maintaining core body temperature across varying environmental conditions by regulating vascular dilation and sweating. Significant dysfunction in these channels may result in hyperthermia or hypothermia as well as pain and pruritus (Wang and Siemens, 2015; Fernández-Carvajal et al., 2022). Notably, among the TRP channel family, thermosensory channels such as TRPV1, TRPM8, and TRPA1 have been identified as key contributors to the development and persistence of CIPN (Villalba-Riquelme et al., 2022; 2025).

Accumulated evidence indicates that TRPV1 plays a significant role in CIPN caused by taxanes, platinum agents, and bortezomib. Exposure of neurons to paclitaxel for 24 h resulted in a marked increase in both TRPV1 expression and function (Villalba-Riquelme et al., 2022). Similarly, a 48-h exposure to oxaliplatin led to a notable elevation in TRPV1 transcriptional expression as well as capsaicin-evoked currents and action potentials (Villalba-Riquelme et al., 2025). Extending these observations, Lamberti et al. reported that paclitaxel exposure first enhanced TRPV1 expression and function and, after a second exposure, subsequently increased TRPA1 and TRPM8 expression levels and function (Lamberti et al., 2026). These latter changes were accompanied by persistent axonal retraction and enhanced peripheral responses to capsaicin and WS12. Furthermore, a recent clinical study demonstrated that daily application of a topical nociceutical formulation containing a TRPV1 soft antagonist delayed the onset of neuropathy and mitigated the severity of neuropathic symptoms (Servitja et al., 2025).

TRPA1 and TRPM8 are cold-activated channels located on small sensory fibers, which have been associated with CIPN pain. Oxaliplatin has been shown to cause acute cold neuropathy by inducing oxidative and metabolic changes that increase the activity of TRPA1 channels in peripheral nerves, while TRPM8 is upregulated in primary nociceptor cultures following 24 h of paclitaxel exposure (Villalba-Riquelme et al., 2022; 2025). In animal studies, inhibition or knockout of TRPA1 eliminates oxaliplatin-induced cold hypersensitivity and reduces paclitaxel-related mechanical allodynia, consistent with a mediating role of TRPA1 in CIPN pain (Nassini et al., 2011; Zhao et al., 2012).

TRPV4 (a mechanosensitive channel) is also upregulated by certain chemotherapies like paclitaxel, potentially contributing to CIPN symptoms (Materazzi et al., 2012). In summary, TRP channels serve as key downstream effectors of chemotherapy-induced nerve hyperexcitability, and they represent attractive targets for neuroprotective or analgesic interventions.

Evidence supporting a role for mechanosensitive PIEZO channels in CIPN is more limited. However, vincristine was shown to potentiate PIEZO2 rapidly adapting mechanically-activating currents in DRG neurons, and knockdown or inhibition of this channel diminished mechanical hypersensitivity in rats (Duan et al., 2023).

The mechanisms underlying ion channel alterations by chemotherapeutic agents are complex and generally opaque. Chemotherapeutic drugs may indirectly modulate ion channel function via modulation of signaling pathways in DRG neurons and/or by indirectly sensitizing them. Consistent with this idea, cumulative paclitaxel exposure increased depolarizing spontaneous fluctuations and input resistance (in a repeated-dose paclitaxel model generated using adult mouse nociceptors), and it produced a stepwise pattern of channel dysregulation characterized by early Na_V_1.8/TRPV1 involvement, and (after the second exposure) subsequent TRPA1, TRPM8, and K_v_3.4 changes (Lamberti et al., 2026). Additionally, transcriptional up- or downregulation of ion channels in response to different chemotherapeutic drugs has been described in multiple studies (Descoeur et al., 2011; Zhang and Dougherty, 2014; Villalba-Riquelme et al., 2025). Increased vesicular trafficking of Na_V_1.7 and Na_V_1.8 channels to the plasma membrane has also been associated with paclitaxel-induced pain symptoms (Baker and Nassar, 2020; Akin et al., 2021).

Another important mechanism of sensitization involves the alteration of proteins that directly interact with these ion channels. For instance, TLR-4 activation directly sensitized TRPV1 following paclitaxel treatment (Li et al., 2014), and sigma-1 receptor antagonists prevented oxaliplatin painful symptoms through interaction with TRPA1 (Marcotti et al., 2023). Activation of immune responses by chemotherapeutic drugs may further sensitize nociceptors. Elevated cytokines such as tumor necrosis factor-α (TNF-α) and interleukins are common responses to chemotherapy, and have been implicated in vincristine-induced pain hypersensitivity via TRPV1 in rats (Wang et al., 2018).

In addition, chemotherapeutic drugs such as thalidomide can induce pain through the generation of oxidative stress byproducts, like hydrogen peroxide, which activate TRPA1 and TRPV4 receptors (De Logu et al., 2020). Activation of protein kinases A and C through proteinase-activated receptor two might further sensitize TRPV1, TRPA1, and TRPV4 in paclitaxel-induced neuropathic pain (Chen et al., 2011), suggesting that post-translational modifications such as ion channel phosphorylation could also significantly contribute to their increased functionality. Furthermore, sex hormones can also modulate the activity of TRP channels in a sex-dependent manner, either enhancing or reducing their activity (Cabañero et al., 2022).

## 7 Major knowledge gaps

The pharmacological toolbox available for preventing and/or treating CIPN is inadequate and, considering the significant advances gained using CIPN preclinical models, can be considered disappointing. The translational bottlenecks are outlined below, and these should be considered in the light of the main strengths and limitations of the major preclinical research pillars used in CIPN studies - murine *in vivo* models, human *in vitro* platforms, and computational *in silico* approaches-as shown in Figure 1.

We next highlight some of the major gaps that may be restraining the translation of the findings to the patients.

### 7.1 Incomplete mechanistic understanding

The interactions between the various injury pathways in CIPN, including axon degeneration, ion channel alterations, and inflammation, are not yet fully elucidated. Because the extents to which axonal degeneration, channelopathy, or immune-mediated damage contribute to the clinical syndrome likely vary depending on both the specific drug used and individual patient factors, reliable biomarkers of these processes are needed to accurately assess their contributions. However, reliable biomarkers are not available for these processes, limiting our ability to assess both these processes and their interactions.

### 7.2 Current gaps in ion channel contribution

The role of ion channel remodelling in CIPN is increasingly being investigated as it may offer a mechanistic basis for symptoms such as burning and tingling sensations, even when there is no measurable nerve fibre loss. There are ongoing questions about how channel modifications interact with structural neuropathy to influence clinical outcomes. It is not fully established why individuals with CIPN display varying symptom profiles, with some experiencing painful, hyperexcitable neuropathy (possibly connected to increased ion channel activity) and others exhibiting numbness and sensory loss (potentially related to axonal degeneration) (Loprinzi et al., 2020). Current research is examining the thresholds and progression of channel plasticity in human nerves, including whether chemotherapy-induced channel changes are reversible or persistent, and if they contribute to chronic pain after treatment. Studies are also exploring potential central nervous system contributions, such as disinhibition in the spinal dorsal horn linked to altered chloride channel function or brain plasticity associated with chronic pain (Yeo et al., 2022). However, additional evidence of the involvement of these processes is required. While ion channel dysfunction can be considered to play an important role in the pathophysiology of CIPN, further research is required to identify specific channels and modulators for preventive or therapeutic strategies.

### 7.3 Variability in patients’ responses

The factors contributing to differences in susceptibility to CIPN are not fully understood; as discussed above, some patients experience pain and hypersensitivity, consistent with neuronal hyperexcitability and small-fiber dysfunction, while other patients experience numbness and sensory loss, consistent with axon loss (Loprinzi et al., 2020). Genome-wide association studies have associated CIPN risk with polymorphisms in several genes required for ion channel function, neuronal repair, and drug metabolism (Argyriou et al., 2006; Rodwin et al., 2022). Patterns of recovery also vary; some individuals regain nerve function, whereas others exhibit persistent deficits. These uncertainties complicate the development of targeted therapies, as it is not yet clear whether degeneration, channel dysfunction, or inflammation should be prioritized in treatment strategies.

### 7.4 Absence of predictive biomarkers for CIPN risk

At present, clinicians lack reliable methods to predict which patients are likely to develop severe CIPN prior to initiating neurotoxic chemotherapy. Beyond general risk factors such as diabetes or cumulative drug dosage, no validated test exists to accurately identify high-risk individuals. Recent research involving breast cancer patients suggested that baseline C-reactive protein levels above 3.91 mg/dL, a body mass index greater than 21.85 kg/m^2^, and unmarried marital status were associated with a 100% predicted probability of CIPN occurrence (Wiranata et al., 2024). While various candidate pharmacogenomic markers have been investigated - for example, a SNP in the CEP72 gene correlating with increased vincristine-induced neuropathy in pediatric leukemia (Christofyllakis et al., 2024) and certain GSTP1 variants linked to oxaliplatin neuropathy (Chen et al., 2010), none have yet been incorporated into routine clinical practice. To date, no genetic or serum biomarker demonstrates sufficient sensitivity or specificity to inform treatment decisions. This lack of predictive biomarkers remains a significant limitation, as it impedes the personalization of chemotherapy regimens. Identifying patients at higher risk for CIPN would enable the consideration of alternative treatments or preventive strategies; currently, oncologists must prescribe neurotoxic agents without clear insight into individual susceptibility to potentially debilitating neuropathy.

### 7.5 Lack of established preventive therapy

Clinical trials have not identified any medication or supplement that consistently prevents CIPN. Randomized studies involving vitamins (E, B12), glutathione, calcium/magnesium infusions, acetyl-L-carnitine, allopurinol, omega-3 fatty acids, cannabinoids, and other agents have generally shown negative or inconclusive outcomes (Loprinzi et al., 2020). Currently, dose management -(that is, limiting or avoiding exposure to the neurotoxic agent-is the only proven approach for reducing CIPN risk. However, this approach can seriously impact cancer treatment efficacy. It is perhaps perplexing (given the many clinical trials already completed) that no prophylactic treatments are available for patients beginning neurotoxic chemotherapy. The lack of positive findings in previous studies may indicate that earlier trials did not address the most relevant mechanisms or that treatment timings were not optimal (and earlier administration prior to the initial nerve injury is required). It is also possible that effective prevention may require a combination of interventions. Notably, a topical nociceutical formulation containing a soft TRPV1 antagonist delayed the appearance of CIPN during chemotherapy and improved the QoL of cancer patients (Servitja et al., 2025). Further research is needed to clarify mechanisms and identify effective strategies for the prevention of CIPN in oncology supportive care.

### 7.6 Limited treatment options for established CIPN

Treatment options for neuropathy are currently limited. Duloxetine, a serotonin-norepinephrine reuptake inhibitor antidepressant, is the only therapy with strong supporting evidence for painful CIPN (Loprinzi et al., 2020). In placebo-controlled trials, duloxetine has demonstrated modest effectiveness in relieving CIPN pain and is recommended as a first-line management option for CIPN-related neuropathic pain (Smith et al., 2013) Typically, duloxetine reduces pain by approximately 30% and does not address numbness. Other medications for neuropathic pain such as gabapentin, pregabalin, and tricyclic antidepressants - often used in diabetes or post-herpetic neuralgia - have shown inconsistent or negative results in CIPN studies (Loprinzi et al., 2020). Moreover, opioid analgesics generally have minimal impact on the tingling or numbness associated with CIPN (Mezzanotte et al., 2022). It should also be noted that there are no drugs currently available to reverse CIPN-associated nerve damage or to promote the regeneration of chemotherapy-damaged peripheral nerves. While experimental treatments, including nerve growth factor modulators and cannabinoids, are under investigation, no therapies for repairing or curing CIPN have yet been approved by the FDA (ClinicalTrials.org, NCT07016971).

Notably, previous clinical trials have largely focused on non-pharmacological interventions, and relatively few have investigated ion channel-targeted therapies. This can be explained by the current limited availability of analgesic drugs (targeting ion channels) and the safety challenges associated with developing new ones. General non-pharmacological measures included in the European Society for Medical Oncology clinical practice guidelines include the application of warm or cold depending on the chemotherapeutic used, as well as the avoidance of extreme temperatures. For instance, patients receiving oxaliplatin are advised to protect themselves from cold by wearing warm shoes and socks, using gloves when handling objects from the fridge or freezer, protecting the ears and nose, and covering the mouth with a scarf (Jordan et al., 2020). Furthermore, two actively recruiting clinical trials are investigating the use of cryotherapy in the hands for patients with taxane-induced neuropathy (ClinicalTrials.org: NCT05928429, NCT06464536). Other non-pharmacological measures that may reduce CIPN symptoms include physical exercise, electroencephalogram-based neurofeedback, and, for patients with refractory neuropathic pain, spinal cord stimulation (Jordan et al., 2020). To date, there is insufficient evidence regarding the use of acupuncture in patients with CIPN; however, seven clinical trials are currently recruiting patients to investigate acupuncture-related interventions for CIPN (ClinicalTrials.org: NCT07377279, NCT06858709, NCT07092111, NCT06769061, NCT07501663, NCT06492083, NCT07177820).

Among ion channel-targeted therapies, TRPV1 is receiving increasing attention. A recent randomized clinical study indicated that a nociceutical formulation targeting palmar epidermal TRPV1 may reduce the incidence of CIPN symptoms in patients’ hands, increasing wellbeing (Servitja et al., 2025). Similarly, patients treated with topical patches containing the TRPV1 agonist capsaicin reported a significant reduction in pain symptoms (Anand et al., 2019). Furthermore, a new clinical trial comparing a 179 mg capsaicin patch with oral duloxetine is currently recruiting patients (ClinicalTrials.org NCT05840562). Topical application of 1% menthol, whose primary molecular sensor is TRPM8, has also been reported to mitigate cancer-related neuropathic pain (Fallon et al., 2015).

Beyond TRP channels, the Na_V_ channel blocker lidocaine has been tested intravenously in CIPN patients, where it demonstrates analgesic effects with moderate long-term benefits (Heuvel et al., 2017). Likewise, a 5% transdermal lidocaine patch significantly improved oxaliplatin-induced neuropathy symptoms in clinical trials (Tsai et al., 2023). In addition, a new clinical trial will investigate a calcium channel blocker, VMD-3866, in a topical gel formulation (ClinicalTrials.org, NCT07072468).

Because supporting clinical evidence is limited, none of these potential therapies are included in current clinical guidelines (e.g., ASCO guidelines). However, it is worth noting that duloxetine, the only treatment currently recommended, has been demonstrated to reduce TRPV1 upregulation in rats, providing further support for the targeting of ion channels in CIPN-related pain. (Wang et al., 2022).

Because of these shortcomings, individuals with chronic CIPN primarily use symptomatic measures and lifestyle modifications to manage their condition, highlighting the ongoing need for new therapies targeting the underlying causes of CIPN.

### 7.7 Translational gap between preclinical and clinical findings

A significant disconnect exists between the promising results achieved in preclinical research and the development of effective clinical therapies for CIPN. Indeed, numerous interventions that have demonstrated efficacy in animal models of CIPN have not produced meaningful benefits in human clinical trials. For instance, while compounds such as acetyl-L-carnitine, nimodipine, and glutamine elicited a reduction in neuropathy in rodent studies, they did not yield substantial outcomes for patients (Ghirardi et al., 2005; Loprinzi et al., 2020). This inconsistency raises questions regarding the validity of current animal models: Do rodent models of CIPN faithfully replicate the chronic and complex neuropathy experienced by patients, or do they primarily capture acute pain behaviors that do not comprehensively reflect patient symptoms such as numbness and functional impairment? It is possible that some preclinical investigations focused on mechanisms - such as acute inflammation or oxidative stress - that may not be central to human CIPN, leading to unsuccessful clinical translation. The persistence of this translational gap impedes drug development, as repeated clinical trial failures may discourage further research despite strong preclinical data. Addressing this issue will likely require refinement of disease models, including greater use of human-derived systems, and improved alignment between preclinical endpoints and clinical outcomes.

### 7.8 Inadequate clinical assessment tools

A practical challenge in CIPN therapy development is the absence of sensitive, standardized methods for neuropathy assessment. In both clinical trials and practice, CIPN is generally evaluated using clinician-rated toxicity scales such as the NCI-CTCAE or through patient surveys; however, each approach has certain limitations. The CTCAE grading offers a broad scale and may not always capture the full extent of patient symptoms, while patient-reported outcome (PRO) questionnaires (e.g., EORTC QLQ-CIPN20) document symptoms but are not universally implemented (Cancer Institute, 2017; Li et al., 2024a). At present, there is no straightforward objective measure or biomarker that reflects CIPN severity - no laboratory test or nerve imaging directly correlates with neuropathy grade. Developing a quantitative assessment tool, such as a handheld device for measuring vibration sensation or a serum biomarker indicating nerve injury, could facilitate improvements in both clinical care and research. Currently, assessing outcomes for CIPN can involve considerable variability and subjectivity, which may obscure the effects of potential treatments in trials. Advancing CIPN assessment, whether through more robust PRO instruments, objective sensor-based approaches, or biomarkers like neurofilament light chain, remains an area for further study (Cerrone et al., 2025).

### 7.9 Psychosocial and long-term impact

Most CIPN research has concentrated on biological mechanisms, with less focus on psychosocial aspects. Chronic CIPN may result in depression, anxiety, and reduced social engagement; for example, a patient experiencing numbness in their feet may have decreased confidence in driving or walking, which can affect independence and participation in social activities (Rahman et al., 2025). The psychological effects of persistent neuropathy are substantial, but interventions addressing mental and functional coping are not extensively studied. Exercise, mindfulness, and cognitive behavioral strategies have been suggested as potentially helpful, and some guidelines recommend these methods (Gowin et al., 2025). Nonetheless, there is limited evidence from rigorous studies evaluating rehabilitative or psychosocial interventions for CIPN. Further research into multidisciplinary management - including physical therapy, occupational therapy, and emotional support - is warranted to better address quality of life for those affected.

### 7.10 Sex differences in CIPN

The potential for sex-related variations in the incidence or presentation of CIPN remains an open question. Some studies suggest that females may experience CIPN more frequently or with greater severity, while other research reports no significant gender differences (Cabañero et al., 2022). Preclinical investigations into pain mechanisms have identified immune-mediated pathways that vary between males and females, which may also contribute to differences in CIPN (Luo et al., 2021). Despite these observations, the topic is underexplored, and most clinical trials do not stratify outcomes by sex. Enhanced understanding of sex-based differences in CIPN would facilitate more individualized management approaches, such as targeted preventive strategies for higher-risk groups, and promote equitable representation in future therapeutic research.

## 8 Outlook and clinical impact

Researchers are developing improved and complementary murine models to more accurately represent the neuropathy and pain symptoms observed in patients. Some refinements could involve using older animals, as advanced age is associated with increased risk of severe CIPN in patients, and assessing the long-term effects of chemotherapeutic drugs, including their combination or cumulative doses, for greater clinical relevance (Yan et al., 2026). Another approach includes modeling comorbidities For instance, paclitaxel treatment potentiates neuropathic cold hypersensitivity in streptozotocin-induced diabetic rodents, supporting the concept that metabolic and chemotherapeutic risk factors can interact to exacerbate peripheral neuropathy (Barrière et al., 2012). Studying the effects of chemotherapeutic drugs on cancer models may further improve translational relevance, as cancer can also alter the functioning of sensory neurons (Haroun et al., 2022).

In parallel, behavioral testing methods are also advancing. These changes include monitoring spontaneous rodent behaviors related to pain (e.g., wheel running or gait analysis), grimace scales, and utilizing automated pain scoring systems to detect ongoing discomfort beyond simple reflexive responses. Novel AI-based algorithms are being used for detecting spontaneous discomfort in animals developing the neuropathy (Zhang et al., 2022; Bak et al., 2024).

Enhancing *in vitro* models to more accurately mimic physiological conditions is essential for advancing CIPN research and improving the translational relevance of experimental results. Extending the duration of primary nociceptor cultures to use long-term *in vitro* models may enable researchers to examine the prolonged or cyclic exposure effects of these drugs on sensory neurons, along with the potential resolution inter-cycle or after treatment (Villalba-Riquelme et al., 2025). Additionally, utilizing human sensory neurons transdifferentiated from aged donor cells, such as fibroblasts, represents a promising strategy. In contrast to human iPSC (hiPSC)-SNs, these cells may preserve epigenetic characteristics linked to CIPN and thus present new opportunities for personalized medicine (Chambers et al., 2012; Blanchard et al., 2015).

The development of coculture systems that integrate DRG neurons with skin-derived cells, immune cells, endothelial cells, and cancer cells holds promise for more effectively modelling the complex multicellular interactions characteristic of this disorder (Ahn et al., 2023; Giorgi et al., 2023). Progress in this area will depend on advances in microfluidic device design to enable compartmentalized primary cultures. As a result, emerging trends include the creation of organoids and 3D cultures, which are now being implemented for the study of CIPN (Rountree et al., 2025). Another interesting approach is the used of human assembloids that reproduce the ascending and descending pain pathways as they can better reflect both peripheral and central sensitization and explore the therapeutic activity of potential drugs (Kim J. I. et al., 2025). However, despite these technological advances, such models remain largely inaccessible due to high costs and limited reproducibility.

Preclinical models commonly identify widespread changes across numerous proteins, but determining which proteins are most relevant to pain symptoms remains difficult. Knockout mice are useful for investigating the functions of individual proteins in pain behavior; however, these studies are resource-intensive and time-consuming. Computational methods may facilitate this research by enabling the screening and prioritization of candidate proteins. In the future, such models may also incorporate patient-derived data, including disease-specific factors like those seen in diabetic neuropathy (Gu et al., 2021). Currently, this area of research is in its early stages and not yet applicable in clinical settings.

Computational models also offer significant value in predicting drug-related toxicities, including hERG cardiotoxicity and drug-induced liver injury (Amorim et al., 2024), and may also be helpful for drug repositioning. Bloomingdale et al. used network simulations to identify potential treatment strategies for bortezomib-induced peripheral neuropathy (BIPN), finding that dexanabinol prevented the mechanical allodynia and hyperalgesia developed in a rat model of BIPN (Bloomingdale et al., 2022). However, current *in silico* approaches are unable to forecast the onset of CIPN. Advancements in machine learning methods and decision algorithms, such as classification and regression trees (CART), are enhancing the identification of high-risk predictors for CIPN. These developments may serve as a foundation for future *in silico* prediction models (Wiranata et al., 2024; Kim J. I. et al., 2025).

Additionally, computational modelling has significant potential for the identification of therapeutic targets tailored to specific phenotypes. For instance, a recent study utilized computational approaches to simulate the spontaneous activity (SA) of nociceptors exposed to paclitaxel, highlighting Na_V_1.8 and delayed rectifier potassium channels (K_DR_) as central molecular determinants (Verma et al., 2020). These targets have likewise been identified *in vitro* preclinical models examining nociceptor responses to paclitaxel and oxaliplatin (Villalba-Riquelme et al., 2022; 2025). Consequently, computational methodologies offer a robust means to screen and prioritize drug targets that are most relevant to specific phenotypes, such as increased SA.

Recent advancements in computational models are poised to significantly influence future research, serving as valuable complements to both *in vitro* and *in vivo* studies. The overarching objective of this approach is to ensure that drug candidates identified in laboratory settings yield tangible clinical benefits, specifically by alleviating and/or preventing chemotherapy-induced peripheral neuropathy. By combining clinical trials, animal models, human *in vitro* systems, and computational modeling researchers will be able to cross-validate individual results and address translational challenges. This integrated approach overcomes the limitations of individual models, offering a more comprehensive and nuanced understanding of CIPN, and ultimately improving survival rates and quality of life for oncology patients.

All preclinical and computational strategies must be clearly aligned with the clinical needs of enhancing the translation of preclinical findings. Clinical research offers critical insights into predictive and progression biomarkers of CIPN. Human-based studies, including imaging and techniques such as nerve conduction studies, further contribute valuable information regarding CIPN pathophysiology (Park et al., 2009). Given the inherent limitations of clinical research, with only a limited number of features accessible for examination, animal models remain indispensable in CIPN investigations by facilitating mechanistic discoveries and preliminary drug testing to ensure safety. Nevertheless, recognition of the limitations intrinsic to animal models has led to the expansion of methodological approaches, including the development of human *in vitro* systems that help validate and enhance findings from animal studies within a human context. Additionally, understanding the contributions of non-neuronal and immune cells to CIPN underscores the importance of advancing these models towards organoids and coculture systems. Drawing upon both clinical and preclinical evidence, computational modeling plays a growing role in screening therapeutic targets and analgesics, as well as in identifying predictors of CIPN to inform clinical decision-making, pharmaceutical research, and preclinical studies. *In silico* approaches can integrate multi-scale data - from ion channel function to drug dosage - to enhance predictions of neurotoxicity.

## 9 Conclusion

In conclusion, although significant advancements have been made over the past decade in understanding the complex mechanisms underlying CIPN, an effective therapeutic intervention remains elusive. This is due not only to the multifactorial and pleiotropic nature of the condition, but also to the limitations of preclinical models that do not fully replicate all aspects of CIPN. A more holistic approach—potentially incorporating topically applied combinations of agents aimed at protecting peripheral nociceptive endings (nociceuticals) - may serve as a valuable adjunct during and after treatment, providing supportive care for patients with neuropathy while new therapies are developed or personalized cancer treatments help reduce CIPN incidence. Importantly, with increasing life expectancy among cancer patients, the integration of supportive care measures during treatment is essential for preserving patient wellbeing. Considering these factors, progress in the development of new therapeutic interventions for CIPN is essential to ensure patient QoL is enhanced.

## References

[B1] Acklin S. Xia F. (2021). The role of nucleotide excision repair in cisplatin-induced peripheral neuropathy: mechanism, prevention, and treatment. Int. J. Mol. Sci. 22, 1975. 10.3390/ijms22041975 33671279 PMC7921932

[B2] Ahn J. Ohk K. Won J. Choi D. H. Jung Y. H. Yang J. H. (2023). Modeling of three-dimensional innervated epidermal like-layer in a microfluidic chip-based coculture system. Nat. Commun. 14, 1488. 10.1038/s41467-023-37187-4 36932093 PMC10023681

[B3] Akin E. J. Alsaloum M. Higerd G. P. Liu S. Zhao P. Dib-Hajj F. B. (2021). Paclitaxel increases axonal localization and vesicular trafficking of Nav1.7. Brain 144, 1727–1737. 10.1093/brain/awab113 33734317 PMC8320304

[B4] Alberts D. S. Noel J. K. (1995). Cisplatin-associated neurotoxicity: can it be prevented? Anticancer Drugs 6, 369–383. 10.1097/00001813-199506000-00003 7670134

[B5] Amorim A. M. B. Piochi L. F. Gaspar A. T. Preto A. J. Rosário-Ferreira N. Moreira I. S. (2024). Advancing drug safety in drug development: bridging computational predictions for enhanced toxicity prediction. Chem. Res. Toxicol. 37, 827–849. 10.1021/acs.chemrestox.3c00352 38758610 PMC11187637

[B6] Anand P. Elsafa E. Privitera R. Naidoo K. Yiangou Y. Donatien P. (2019). Rational treatment of chemotherapy-induced peripheral neuropathy with capsaicin 8% patch: from pain relief towards disease modification. J. Pain Res. 12, 2039–2052. 10.2147/JPR.S213912 31308732 PMC6613356

[B7] Argyriou A. A. Chroni E. Koutras A. Iconomou G. Papapetropoulos S. Polychronopoulos P. (2006). Preventing paclitaxel-induced peripheral neuropathy: a phase II trial of vitamin E supplementation. J. Pain Symptom Manage. 32, 237–244. 10.1016/j.jpainsymman.2006.03.013 16939848

[B8] Bak M. S. Park H. Yoon H. Chung G. Shin H. Shin S. (2024). Machine learning-based evaluation of spontaneous pain and analgesics from cellular calcium signals in the mouse primary somatosensory cortex using explainable features. Front. Mol. Neurosci. 17, 1356453. 10.3389/fnmol.2024.1356453 38450042 PMC10915002

[B9] Baker M. D. Nassar M. A. (2020). Painful and painless mutations of SCN9A and SCN11A voltage-gated sodium channels. Pflugers Arch. 472, 865–880. 10.1007/s00424-020-02419-9 32601768 PMC7351857

[B10] Barrière D. A. Rieusset J. Chanteranne D. Busserolles J. Chauvin M.-A. Chapuis L. (2012). Paclitaxel therapy potentiates cold hyperalgesia in streptozotocin-induced diabetic rats through enhanced mitochondrial reactive oxygen species production and TRPA1 sensitization. Pain 153 (3), 553–561. 10.1016/j.pain.2011.11.019 22177224

[B11] Battaglini E. Goldstein D. Grimison P. McCullough S. Mendoza-Jones P. Park S. B. (2021). Chemotherapy-induced peripheral neurotoxicity in cancer survivors: predictors of long-term patient outcomes. JNCCN J. Natl. Compr. Cancer Netw. 19, 821–828. 10.6004/jnccn.2021.7026 34340206

[B12] Bechakra M. Nieuwenhoff M. D. van Rosmalen J. Groeneveld G. J. Scheltens-de Boer M. Sonneveld P. (2018). Clinical, electrophysiological, and cutaneous innervation changes in patients with bortezomib-induced peripheral neuropathy reveal insight into mechanisms of neuropathic pain. Mol. Pain 14, 1744806918797042. 10.1177/1744806918797042 30152246 PMC6113731

[B13] Bernges F. Holler E. (1991). The reaction of platinum(II) complexes with DNA. Kinetics of intrastrand crosslink formation *in vitro* . Nucleic Acids Res. 19 (7), 1483–1489. PMID: 2027756; PMCID: PMC333905. 10.1093/nar/19.7.1483 2027756 PMC333905

[B14] Blanchard J. W. Eade K. T. Szůcs A. Lo Sardo V. Tsunemoto R. K. Williams D. (2015). Selective conversion of fibroblasts into peripheral sensory neurons. Nat. Neurosci. 18, 25–35. 10.1038/nn.3887 25420069 PMC4466122

[B15] Bloomingdale P. Meregalli C. Pollard K. Canta A. Chiorazzi A. Fumagalli G. (2022). Systems pharmacology modeling identifies a novel treatment strategy for Bortezomib-Induced neuropathic pain. Front. Pharmacol. 12, 817236. 10.3389/fphar.2021.817236 35126148 PMC8809372

[B16] Bozal S. B. Sjogren G. Costa A. P. Brown J. S. Roberts S. Baker D. (2024). Development of an automated 3D high content cell screening platform for organoid phenotyping. SLAS Discov. 29, 100182. 10.1016/j.slasd.2024.100182 39245180 PMC12380041

[B17] Cabañero D. Villalba-Riquelme E. Fernández-Ballester G. Fernández-Carvajal A. Ferrer-Montiel A. (2022). ThermoTRP channels in pain sexual dimorphism: new insights for drug intervention. Pharmacol. Ther. 240, 108297. 10.1016/j.pharmthera.2022.108297 36202261

[B18] Cancer Institute N. (2017). Common terminology criteria for adverse events (CTCAE) common terminology criteria for adverse events (CTCAE) v5.0. Available online at: https://www.meddra.org/ (Accessed October 25, 2025).

[B19] Carpenter A. E. Jones T. R. Lamprecht M. R. Clarke C. Kang I. H. Friman O. (2006). CellProfiler: image analysis software for identifying and quantifying cell phenotypes. Genome Biol. 7, R100. 10.1186/gb-2006-7-10-r100 17076895 PMC1794559

[B20] Cashman C. R. Höke A. (2015). Mechanisms of distal axonal degeneration in peripheral neuropathies. Neurosci. Lett. 596, 33–50. 10.1016/j.neulet.2015.01.048 25617478 PMC4428955

[B21] Cayre M. Falque M. Mercier O. Magalon K. Durbec P. (2021). Myelin repair: from animal models to humans. Front. Cell. Neurosci. 15, 604865. 10.3389/fncel.2021.604865 33935649 PMC8079744

[B22] Cerrone V. Esposito D. Montedoro M. Andretta V. Leoni M. L. G. Cuomo A. (2025). Diagnosis of chemotherapy-induced peripheral neurotoxicity: a scoping review. Vivo (Brooklyn) 39, 3041–3059. 10.21873/invivo.14107 PMC12588251 41167691

[B23] Chambers S. M. Qi Y. Mica Y. Lee G. Zhang X. J. Niu L. (2012). Combined small-molecule inhibition accelerates developmental timing and converts human pluripotent stem cells into nociceptors. Nat. Biotechnol. 30, 715–720. 10.1038/nbt.2249 22750882 PMC3516136

[B24] Chen Y. C. Tzeng C. H. Chen P. M. Lin J. K. Lin T. C. Chen W. S. (2010). Influence of GSTP1 I105V polymorphism on cumulative neuropathy and outcome of FOLFOX-4 treatment in Asian patients with colorectal carcinoma. Cancer Sci. 101, 530–535. 10.1111/j.1349-7006.2009.01418.x 19922504 PMC11158438

[B25] Chen Y. Yang C. Wang Z. J. (2011). Proteinase-activated receptor 2 sensitizes transient receptor potential vanilloid 1, transient receptor potential vanilloid 4, and transient receptor potential ankyrin 1 in paclitaxel-induced neuropathic pain. Neuroscience 193, 440–451. 10.1016/j.neuroscience.2011.06.085 21763756

[B26] Christofyllakis K. Kaddu-Mulindwa D. Lesan V. Rixecker T. Kos I. A. Held G. (2024). An inherited genetic variant of the CEP72 gene is associated with the development of vincristine-induced peripheral neuropathy in female patients with aggressive B-cell lymphoma. Ann. Hematol. 103, 4599–4606. 10.1007/s00277-024-05973-9 39227453 PMC11534822

[B27] de Lima A. P. N. Zhang H. Chen L. Effraim P. R. Gomis-Perez C. Cheng X. (2024). Nav1.8 in small dorsal root ganglion neurons contributes to vincristine-induced mechanical allodynia. Brain 147, 3157–3170. 10.1093/brain/awae071 38447953

[B28] De Logu F. Trevisan G. Marone I. M. Coppi E. Padilha Dalenogare D. Titiz M. (2020). Oxidative stress mediates thalidomide-induced pain by targeting peripheral TRPA1 and central TRPV4. BMC Biol. 18, 197. 10.1186/s12915-020-00935-9 33317522 PMC7737339

[B29] Derman B. A. Davis A. M. (2021). Recommendations for prevention and management of chemotherapy-induced peripheral neuropathy. JAMA - J. Am. Med. Assoc. 326, 1058. 10.1001/jama.2021.7458 34546311

[B30] Descoeur J. Pereira V. Pizzoccaro A. Francois A. Ling B. Maffre V. (2011). Oxaliplatin-induced cold hypersensitivity is due to remodelling of ion channel expression in nociceptors. EMBO Mol. Med. 3, 266–278. 10.1002/emmm.201100134 21438154 PMC3377073

[B31] Dionisi M. Ruffinatti F. A. Riva B. Lim D. Canta A. Meregalli C. (2020). Early stimulation of trek channel transcription and activity induced by oxaliplatin-dependent cytosolic acidification. Int. J. Mol. Sci. 21. 10.3390/ijms21197164 32998392 PMC7584002

[B32] Duan M. Jia Y. Huo L. Gao Y. Wang J. Zhang W. (2023). Potentiation of PIEZO2 mechanically-activated currents in sensory neurons mediates vincristine-induced mechanical hypersensitivity. Acta Pharm. Sin. B 13 (8), 3365–3381. 10.1016/j.apsb.2023.05.010 37655331 PMC10466006

[B33] Eldridge S. Guo L. Hamre J. (2020). A comparative review of chemotherapy-induced peripheral neuropathy in *in vivo* and *in vitro* models. Toxicol. Pathol. 48, 190–201. 10.1177/0192623319861937 31331249 PMC6917839

[B34] Fallon M. T. Storey D. J. Krishan A. Weir C. J. Mitchell R. Fleetwood-Walker S. M. (2015). Cancer treatment-related neuropathic pain: proof of concept study with Menthol—A TRPM8 agonist. Support. Care Cancer 23, 2769–2777. 10.1007/s00520-015-2642-8 25680765 PMC4519585

[B35] Fernández-Carvajal A. Fernández-Ballester G. Ferrer-Montiel A. (2022). TRPV1 in chronic pruritus and pain: soft modulation as a therapeutic strategy. Front. Mol. Neurosci. 15, 930964. 10.3389/fnmol.2022.930964 36117910 PMC9478410

[B36] Flatters S. J. L. Dougherty P. M. Colvin L. A. (2017). Clinical and preclinical perspectives on chemotherapy-induced peripheral neuropathy (CIPN): a narrative review. Br. J. Anaesth. 119, 737–749. 10.1093/bja/aex229 29121279

[B37] Ghirardi O. Vertechy M. Vesci L. Canta A. Nicolini G. Galbiati S. (2005). Chemotherapy-induced allodinia: neuroprotective effect of acetyl-L-carnitine. Vivo (Brooklyn) 19, 631–637. 15875786

[B38] Giorgi S. Lamberti A. Butrón L. Gross-Amat O. Alarcón-Alarcón D. Rodríguez-Cañas E. (2023). Compartmentalized primary cultures of dorsal root ganglion neurons to model peripheral pathophysiological conditions. Mol. Pain 19, 17448069231197102. 10.1177/17448069231197102 37578145 PMC10521292

[B39] Gowin K. Ahn E. R. Lopez A. M. (2025). Synthesis and educational overview of the ASCO–society of integrative oncology guidelines: on the path to implementation. Am. Soc. Clin. Oncol. Educ. Book 45, e471830. 10.1200/edbk-25-471830 40570266

[B40] Griffin J. W. Pan B. H. Polley M. A. Hoffman P. N. Farah M. H. (2010). Measuring nerve regeneration in the mouse. Exp. Neurol. 223, 60–71. 10.1016/j.expneurol.2009.12.033 20080088

[B41] Grisold W. Cavaletti G. Windebank A. J. (2012). Peripheral neuropathies from chemotherapeutics and targeted agents: diagnosis, treatment, and prevention. Neuro. Oncol. 14, iv45–iv54. 10.1093/neuonc/nos203 23095830 PMC3480245

[B42] Gu J. Lu H. Chen C. Gu Z. Hu M. Liu L. (2021). Diabetes mellitus as a risk factor for chemotherapy-induced peripheral neuropathy: a meta-analysis. Support. Care Cancer 29, 7461–7469. 10.1007/s00520-021-06321-7 34085148 PMC8550712

[B43] Gurwitz D. Weizman A. (2001). Animal models and human genome diversity: the pitfalls of inbred mice. Drug Discov. Today 6 (15), 766–768. 10.1016/S1359-6446(01)01874-8 11470580

[B44] Hammad A. Mohamed M S. A. Khalifa M. El-Daly M. (2023). Mechanisms of Paclitaxel-induced peripheral neuropathy. J. Advanced Biomed. Pharm. Sci. 6, 25–35. 10.21608/jabps.2022.170238.1172

[B45] Han Y. Smith M. T. (2013). Pathobiology of cancer chemotherapy-induced peripheral neuropathy (CIPN). Front. Pharmacol. 4 (DEC), 156. 10.3389/fphar.2013.00156 24385965 PMC3866393

[B46] Han X. Matsuda N. Yamanaka M. Suzuki I. (2024). Development of a novel microphysiological system for peripheral neurotoxicity prediction using human iPSC-Derived neurons with morphological deep learning. Toxics 12, 809. 10.3390/toxics12110809 39590989 PMC11598741

[B47] Haroun R. Wood J. N. Sikandar S. (2022). Mechanisms of cancer pain. Front. Pain Res. 3, 1030899. 10.3389/fpain.2022.1030899 PMC9845956 36688083

[B48] Hershman D. L. Lacchetti C. Dworkin R. H. Lavoie Smith E. M. Bleeker J. Cavaletti G. (2014). Prevention and management of chemotherapy-induced peripheral neuropathy in survivors of adult cancers: american society of clinical oncology clinical practice guideline. J. Clin. Oncol. 32, 1941–1967. 10.1200/JCO.2013.54.0914 24733808

[B49] Heuvel S. A. S. V. D. Wal S. E. I. V. D. Smedes L. A. Radema S. A. Alfen N. V. Vissers K. C. P. (2017). Intravenous lidocaine: old-school drug, new purpose-reduction of intractable pain in patients with chemotherapy induced peripheral neuropathy. Pain Res. Manag. 2017, 2017–2019. 10.1155/2017/8053474 PMC5387833 28458593

[B50] Holzer A. K. Karreman C. Suciu I. Furmanowsky L. S. Wohlfarth H. Loser D. (2022). Generation of human nociceptor-enriched sensory neurons for the study of pain-related dysfunctions. Stem Cells Transl. Med. 11, 727–741. 10.1093/stcltm/szac031 35689659 PMC9299516

[B51] Iseppon F. Luiz A. P. Linley J. E. Wood J. N. (2023). Pregabalin silences oxaliplatin-activated sensory neurons to relieve cold allodynia. eNeuro 10, ENEURO.0395–22.2022. 10.1523/ENEURO.0395-22.2022 36720644 PMC9998121

[B52] Jolivalt C. G. Frizzi K. E. Guernsey L. Marquez A. Ochoa J. Rodriguez M. (2016). Peripheral neuropathy in mouse models of diabetes. Curr. Protoc. Mouse Biol. 6, 223–255. 10.1002/cpmo.11 27584552 PMC5023323

[B53] Jones J. Correll D. J. Lechner S. M. Jazic I. Miao X. Shaw D. (2023). Selective inhibition of Na V 1.8 with VX-548 for acute pain. N. Engl. J. Med. 389, 393–405. 10.1056/nejmoa2209870 37530822

[B54] Jordan B. Margulies A. Cardoso F. Cavaletti G. Haugnes H. S. Jahn P. (2020). Systemic anticancer therapy-induced peripheral and central neurotoxicity: ESMO-EONS-EANO clinical practice guidelines for diagnosis, prevention, treatment and follow-up. Ann. Oncol. 31 (10), 1306–1319. 10.1016/j.annonc.2020.07.003 32739407

[B55] Kalia A. K. Rösseler C. Granja-Vazquez R. Ahmad A. Pancrazio J. J. Neureiter A. (2024). How to differentiate induced pluripotent stem cells into sensory neurons for disease modelling: a functional assessment. Stem Cell. Res. Ther. 15, 99. 10.1186/s13287-024-03696-2 38581069 PMC10998320

[B56] Kang L. Tian Y. Xu S. Chen H. (2021). Oxaliplatin-induced peripheral neuropathy: clinical features, mechanisms, prevention and treatment. J. Neurol. 268, 3269–3282. 10.1007/s00415-020-09942-w 32474658

[B57] Kerckhove N. Collin A. Condé S. Chaleteix C. Pezet D. Balayssac D. (2017). Long-term effects, pathophysiological mechanisms, and risk factors of chemotherapy-induced peripheral neuropathies: a comprehensive literature review. Front. Pharmacol. 8, 86. 10.3389/fphar.2017.00086 28286483 PMC5323411

[B58] Kim, H. K. Xing J. Jung Y. S. Park J.Il Kim H. Y. Kim J. (2025). Exacerbation of paclitaxel-induced neuropathic pain behaviors in breast tumor–bearing mice. Mol. Pain 21, 17448069251380034. 10.1177/17448069251380034 40913249 PMC12536134

[B59] Kim, J. I. Imaizumi K. Jurjuț O. Kelley K. W. Wang D. Thete M. V. (2025). Human assembloid model of the ascending neural sensory pathway. Nature 642, 143–153. 10.1038/s41586-025-08808-3 40205039 PMC12137141

[B60] Kramer L. Nguyen H. T. Jacobs E. McCoy L. Lowry Curley J. Sharma A. D. (2020). Modeling chemotherapy-induced peripheral neuropathy using a nerve-on-a-chip microphysiological system. ALTEX 37, 350–364. 10.14573/altex.2001181 32388569

[B61] Lamberti A. Lozano V. M. Fernández-Carvajal A. Ferrer-Montiel A. (2026). Persistent *in vitro* nociceptor hyperexcitability and axonal retraction produced by repeated paclitaxel doses. FEBS J., febs.70517. 10.1111/febs.70517 41902442

[B62] Lampart F. L. Iber D. Doumpas N. (2023). Organoids in high-throughput and high-content screenings. Front. Chem. Eng. 5, 1120348. 10.3389/fceng.2023.1120348

[B63] Lewis K. Vallone V. F. Dordevic A. Kern J. Stachelscheid H. Endres M. (2025). Generation of ten human induced pluripotent stem cell lines (hiPSCs) from patients with and without chemotherapy-induced peripheral neuropathy (CIPN) and post chemotherapy cognitive impairment (PCCI). Stem Cell. Res. 84, 103674. 10.1016/j.scr.2025.103674 40015137

[B64] Li Y. Zhang H. Zhang H. Kosturakis A. K. Jawad A. B. Dougherty P. M. (2014). Toll-like receptor 4 signaling contributes to paclitaxel-induced peripheral neuropathy. J. Pain 15, 712–725. 10.1016/j.jpain.2014.04.001 24755282 PMC4083500

[B65] Li Y. Adamek P. Zhang H. Tatsui C. E. Rhines L. D. Mrozkova P. (2015). The cancer chemotherapeutic paclitaxel increases human and rodent sensory neuron responses to TRPV1 by activation of TLR4. J. Neurosci. 35, 13487–13500. 10.1523/JNEUROSCI.1956-15.2015 26424893 PMC4588613

[B66] Li Y. North R. Y. Rhines L. D. Tatsui C. E. Rao G. Edwards D. D. (2018). Drg voltage-gated sodium channel 1.7 is upregulated in paclitaxel-induced neuropathy in rats and in humans with neuropathic pain. J. Neurosci. 38, 1124–1136. 10.1523/JNEUROSCI.0899-17.2017 29255002 PMC5792474

[B67] Li L. Li J. Zuo Y. Dang D. Frost J. A. Yang Q. (2019). Activation of KCNQ channels prevents paclitaxel-induced peripheral neuropathy and associated neuropathic pain. J. Pain 20, 528–539. 10.1016/j.jpain.2018.11.001 30471428 PMC7337983

[B68] Li Y. Marri T. North R. Y. Rhodes H. R. Uhelski M. L. Tatsui C. E. (2021). Chemotherapy-induced peripheral neuropathy in a dish: dorsal root ganglion cells treated *in vitro* with paclitaxel show biochemical and physiological responses parallel to that seen *in vivo* . Pain 162, 84–96. 10.1097/j.pain.0000000000002005 32694383 PMC7744394

[B69] Li T. Timmins H. C. Mahfouz F. M. Trinh T. Mizrahi D. Horvath L. G. (2024a). Validity of patient-reported outcome measures in evaluating nerve damage following chemotherapy. JAMA Netw. Open 7, e2424139. 10.1001/jamanetworkopen.2024.24139 39120903 PMC11316238

[B70] Li T. Trinh T. Bosco A. Kiernan M. C. Goldstein D. Park S. B. (2024b). Characterising vincristine-induced peripheral neuropathy in adults: symptom development and long-term persistent outcomes. Support. Care Cancer 32, 278. 10.1007/s00520-024-08484-5 38592525 PMC11003903

[B71] Lickfett S. Menacho C. Zink A. Telugu N. S. Beller M. Diecke S. (2022). High-content analysis of neuronal morphology in human iPSC-derived neurons. Star. Protoc. 3, 101567. 10.1016/j.xpro.2022.101567 35990743 PMC9386098

[B72] Little A. C. Kovalenko I. Goo L. E. Hong H. S. Kerk S. A. Yates J. A. (2020). High-content fluorescence imaging with the metabolic flux assay reveals insights into mitochondrial properties and functions. Commun. Biol. 3, 271. 10.1038/s42003-020-0988-z 32472013 PMC7260371

[B73] Loprinzi C. L. Maddocks-Christianson K. Wolf S. L. Rao R. D. James P. Dyck B. (2007). The Paclitaxel Acute Pain Syndrome: Sensitization of Nociceptors as the Putative Mechanism. 10.1097/PPO.0b013e31815a999b 18032978

[B74] Loprinzi C. L. Lacchetti C. Bleeker J. Cavaletti G. Chauhan C. Hertz D. L. (2020). Prevention and management of chemotherapy-induced peripheral neuropathy in survivors of adult cancers: ASCO guideline update. J. Clin. Oncol. 38, 3325–3348. 10.1200/JCO.20.01399 32663120

[B75] Luo X. Chen O. Wang Z. Bang S. Ji J. Lee S. H. (2021). IL-23/IL-17A/TRPV1 axis produces mechanical pain *via* macrophage-sensory neuron crosstalk in female mice. Neuron 109, 2691–2706.e5. 10.1016/j.neuron.2021.06.015 34473953 PMC8425601

[B76] Lv X. Mao Y. Cao S. Feng Y. (2023). Animal models of chemotherapy-induced peripheral neuropathy for hematological malignancies: a review. Ibrain 9, 72–89. 10.1002/ibra.12086 37786517 PMC10529012

[B77] Macdonald D. I. Luiz A. P. Iseppon F. Millet Q. Emery E. C. Wood J. N. (2021). Silent cold-sensing neurons contribute to cold allodynia in neuropathic pain. Brain 144, 1711–1726. 10.1093/brain/awab086 33693512 PMC8320254

[B78] Mansooralavi N. Khomula E. V. Levine J. D. (2023). Duloxetine prevents bortezomib and paclitaxel large-fiber chemotherapy-induced peripheral neuropathy (LF-CIPN) in sprague dawley rats. Mol. Pain 19, 17448069231185694. 10.1177/17448069231185694 37338165 PMC10288414

[B79] Marcotti A. Fernández-Trillo J. González A. Vizcaíno-Escoto M. Ros-Arlanzón P. Romero L. (2023). TRPA1 modulation by Sigma-1 receptor prevents oxaliplatin-induced painful peripheral neuropathy. Brain 146, 475–491. 10.1093/brain/awac273 35871491 PMC9924907

[B80] Materazzi S. Fusi C. Benemei S. Pedretti P. Patacchini R. Nilius B. (2012). TRPA1 and TRPV4 mediate paclitaxel-induced peripheral neuropathy in mice via a glutathione-sensitive mechanism. Pflugers Arch. 463, 561–569. 10.1007/s00424-011-1071-x 22258694

[B81] McDowell G. C. Pope J. E. (2016). Intrathecal ziconotide: dosing and administration strategies in patients with refractory chronic pain. Neuromodulation 19, 522–532. 10.1111/ner.12392 26856969 PMC5067570

[B82] McWhinney S. R. Goldberg R. M. McLeod H. L. (2009). Platinum neurotoxicity pharmacogenetics. Mol. Cancer Ther. 8, 10–16. 10.1158/1535-7163.MCT-08-0840 19139108 PMC2651829

[B83] Mertens J. Paquola A. C. M. Ku M. Hatch E. Böhnke L. Ladjevardi S. (2015). Directly reprogrammed human neurons retain aging-associated transcriptomic signatures and reveal age-related nucleocytoplasmic defects. Cell. Stem Cell. 17 (6), 705–718. 10.1016/j.stem.2015.09.001 26456686 PMC5929130

[B84] Mezzanotte J. N. Grimm M. Shinde N. V. Nolan T. Worthen-Chaudhari L. Williams N. O. (2022). Updates in the treatment of chemotherapy-induced peripheral neuropathy. Curr. Treat. Options Oncol. 23 (1), 29–42. 10.1007/s11864-021-00926-0 35167004 PMC9642075

[B85] Mols F. Beijers T. Vreugdenhil G. Van De Poll-Franse L. (2014). Chemotherapy-induced peripheral neuropathy and its association with quality of life: a systematic review. Support. Care Cancer 22, 2261–2269. 10.1007/s00520-014-2255-7 24789421

[B86] Moreau P. Pylypenko H. Grosicki S. Karamanesht I. Leleu X. Grishunina M. (2011). Subcutaneous versus intravenous administration of bortezomib in patients with relapsed multiple myeloma: a randomised, phase 3, non-inferiority study. Lancet. Oncol. 12, 431–471. 10.1016/S1470 21507715

[B87] Mortensen C. Andersen N. E. Stage T. B. (2022). Bridging the translational gap in chemotherapy-induced peripheral neuropathy with iPSC-Based modeling. Cancers (Basel) 14, 3939. 10.3390/cancers14163939 36010931 PMC9406154

[B88] Mortensen C. Thomsen M. T. Chua K. C. Hammer H. S. Nielsen F. Pötz O. (2024). Modeling mechanisms of chemotherapy-induced peripheral neuropathy and chemotherapy transport using induced pluripotent stem cell-derived sensory neurons. Neuropharmacology 258, 110062. 10.1016/j.neuropharm.2024.110062 38972371

[B89] Naji-Esfahani H. Vaseghi G. Safaeian L. Pilehvarian A. A. Abed A. Rafieian-Kopaei M. (2016). Gender differences in a mouse model of chemotherapy-induced neuropathic pain. Lab. Anim. 50, 15–20. 10.1177/0023677215575863 25732574

[B90] Nassini R. Gees M. Harrison S. De Siena G. Materazzi S. Moretto N. (2011). Oxaliplatin elicits mechanical and cold allodynia in rodents via TRPA1 receptor stimulation. Pain 152 (7), 1621–1631. 10.1016/j.pain.2011.02.051 21481532

[B91] Nassini R. Benemei S. Fusi C. Trevisan G. Materazzi S. (2013). Transient receptor potential channels in chemotherapy-induced neuropathy. Open Pain J. 6, 127–136. 10.2174/1876386301306010127

[B92] Park S. Krishnan A. Lin C. Goldstein D. Friedlander M. Kiernan M. (2008). Mechanisms underlying chemotherapy-induced neurotoxicity and the potential for neuroprotective strategies. Curr. Med. Chem. 15, 3081–3094. 10.2174/092986708786848569 19075655

[B93] Park S. B. Goldstein D. Lin C. S. Y. Krishnan A. V. Friedlander M. L. Kiernan M. C. (2009). Acute abnormalities of sensory nerve function associated with oxaliplatin-induced neurotoxicity. J. Clin. Oncol. 27, 1243–1249. 10.1200/JCO.2008.19.3425 19164207

[B94] Park S. B. Goldstein D. Krishnan A. V. Lin C. S. Friedlander M. L. Cassidy J. (2013). Chemotherapy‐induced peripheral neurotoxicity: a critical analysis. CA Cancer J. Clin. 63, 419–437. 10.3322/caac.21204 24590861

[B95] Park S. B. Cetinkaya-Fisgin A. Argyriou A. A. Höke A. Cavaletti G. Alberti P. (2023). Axonal degeneration in chemotherapy-induced peripheral neurotoxicity: clinical and experimental evidence. J. Neurol. Neurosurg. Psychiatry 94, 962–972. 10.1136/jnnp-2021-328323 37015772 PMC10579520

[B96] Pero M. E. Meregalli C. Qu X. Shin G. J. E. Kumar A. Shorey M. (2021). Pathogenic role of Delta 2 tubulin in bortezomib-induced peripheral neuropathy. Proc. Natl. Acad. Sci. U. S. A. 118, e2012685118. 10.1073/pnas.2012685118 33468672 PMC7848563

[B97] Petrova V. Snavely A. R. Splaine J. Zhen S. Singh B. Pandey R. (2024). Identification of novel neuroprotectants against vincristine-induced neurotoxicity in iPSC-derived neurons. Cell. Mol. Life Sci. 81, 315. 10.1007/s00018-024-05340-x 39066803 PMC11335239

[B98] Qi L. Iskols M. Shi D. Reddy P. Walker C. Lezgiyeva K. (2024). A mouse DRG genetic toolkit reveals morphological and physiological diversity of somatosensory neuron subtypes. Cell. 187, 1508–1526.e16. 10.1016/j.cell.2024.02.006 38442711 PMC10947841

[B99] Rahman N. Sukumar J. Lustberg M. B. (2025). Chronic chemotherapy-induced peripheral neuropathy: living with neuropathy during and after cancer treatments. Ann. Palliat. Med. 14, 196–216. 10.21037/apm-24-154 40211744

[B100] Riva B. Dionisi M. Potenzieri A. Chiorazzi A. Cordero-Sanchez C. Rigolio R. (2018). Oxaliplatin induces pH acidification in dorsal root ganglia neurons. Sci. Rep. 8, 15084. 10.1038/s41598-018-33508-6 30305703 PMC6180129

[B101] Rodwin R. L. Siddiq N. Z. Ehrlich B. E. Lustberg M. B. (2022). Biomarkers of chemotherapy-induced peripheral neuropathy: current status and future directions. Front. Pain Res. 3, 864910. 10.3389/fpain.2022.864910 PMC8963873 35360655

[B102] Rountree C. Rodriguez T. Byrne E. Schmidt E. Schwarzbach E. Dillon S. (2025). Human Peripheral Nerve-on-a-Chip on a Multiwell Microelectrode Array as a Scalable Preclinical Neurotoxicity Assay. 10.1101/2025.06.09.658615

[B103] Saif M. W. Reardon J. (2005). Management of oxaliplatin-induced peripheral neuropathy. Ther. Clin. Risk Manag. 1, 249–258. 18360567 PMC1661634

[B104] Salinas N. Nowak E. Etienne M. Legoupil D. Fouchard M. Brenaut E. (2021). Causes of pruritus in patients treated with immune checkpoint inhibitors for melanomas or skin carcinomas. Front. Med. (Lausanne). 8, 632683. 10.3389/fmed.2021.632683 33634154 PMC7900003

[B105] Schinke C. Fernandez Vallone V. Ivanov A. Peng Y. Körtvelyessy P. Nolte L. (2021). Modeling chemotherapy induced neurotoxicity with human induced pluripotent stem cell (iPSC) -derived sensory neurons. Neurobiol. Dis. 155, 105391. 10.1016/j.nbd.2021.105391 33984509

[B106] Schmitt L. I. Leo M. Kleinschnitz C. Hagenacker T. (2018). Oxaliplatin modulates the characteristics of voltage-gated calcium channels and action potentials in small dorsal root ganglion neurons of rats. Mol. Neurobiol. 55, 8842–8855. 10.1007/s12035-018-1029-5 29603093

[B107] Seretny M. Currie G. L. Sena E. S. Ramnarine S. Grant R. Macleod M. R. (2014). Incidence, prevalence, and predictors of chemotherapy-induced peripheral neuropathy: a systematic review and meta-analysis. Pain 155, 2461–2470. 10.1016/j.pain.2014.09.020 25261162

[B108] Servitja S. Castro-Henriques M. Álvarez-Busto I. Díez-Franco C. Medina-Castillo A. Algarra-García M. A. (2025). A topical nociceutical formulation ameliorates chemotherapy-induced peripheral neuropathy: a pilot randomized clinical study. Clin. Transl. Oncol. 28, 1040–1048. 10.1007/s12094-025-04062-1 41032160 PMC12920311

[B109] Shin G. J. E. Pero M. E. Hammond L. A. Burgos A. Kumar A. Galindo S. E. (2021). Integrins protect sensory neurons in models of paclitaxel-induced peripheral sensory neuropathy. Proc. Natl. Acad. Sci. U. S. A. 118, e2006050118. 10.1073/pnas.2006050118 33876743 PMC8053987

[B110] Siegel D. Martin T. Nooka A. Harvey R. D. Vij R. Niesvizky R. (2013). Integrated safety profile of single-agent carfilzomib: experience from 526 patients enrolled in 4 phase II clinical studies. Haematologica 98, 1753–1761. 10.3324/haematol.2013.089334 23935022 PMC3815177

[B111] Smith E. M. L. Pang H. Cirrincione C. Fleishman S. Paskett E. D. Ahles T. (2013). Effect of duloxetine on pain, function, and quality of life among patients with chemotherapy-induced painful peripheral neuropathy: a randomized clinical trial. JAMA 309, 1359–1367. 10.1001/jama.2013.2813 23549581 PMC3912515

[B112] Sogbein O. Paul P. Umar M. Chaari A. Batuman V. Upadhyay R. (2024). Bortezomib in cancer therapy: mechanisms, side effects, and future proteasome inhibitors. Life Sci. 358, 123125. 10.1016/j.lfs.2024.123125 39413903

[B113] Starobova H. Vetter I. (2017). Pathophysiology of chemotherapy-induced peripheral neuropathy. Front. Mol. Neurosci. 10, 174. 10.3389/fnmol.2017.00174 28620280 PMC5450696

[B114] Thulin J. D. Bradfield J. F. Bergdall V. K. Conour L. A. Grady A. W. Hickman D. L. (2014). The cost of self-imposed regulatory burden in animal research. FASEB J. 28, 3297–3300. 10.1096/fj.14-254094 24784580

[B115] Tsai J. H. Liu I. T. Su P. F. Huang Y. T. Chiu G. L. Chen Y. Y. (2023). Lidocaine transdermal patches reduced pain intensity in neuropathic cancer patients already receiving opioid treatment. BMC Palliat. Care 22, 4. 10.1186/s12904-023-01126-3 36609269 PMC9824981

[B116] Uittenboogaard A. Neutel C. L. G. Ket J. C. F. Njuguna F. Huitema A. D. R. Kaspers G. J. L. (2022). Pharmacogenomics of vincristine-induced peripheral neuropathy in children with cancer: a systematic review and meta-analysis. Cancers (Basel) 14, 612. 10.3390/cancers14030612 35158880 PMC8833506

[B117] Verma P. Eaton M. Kienle A. Flockerzi D. Yang Y. Ramkrishna D. (2020). Examining sodium and potassium channel conductances involved in hyperexcitability of chemotherapy-induced peripheral neuropathy: a mathematical and cell culture-based study. Front. Comput. Neurosci. 14, 564980. 10.3389/fncom.2020.564980 33178002 PMC7593680

[B118] Vertex Pharmaceuticals Incorporated (2026). A phase 3, randomized, double-blind, Placebo- and active-controlled study of the efficacy and safety of suzetrigine in subjects with pain associated with diabetic peripheral neuropathy. Ongoing Clinical Trial. ClinicalTrials.gov Identifier NCT06628908.

[B119] Villalba-Riquelme E. de la Torre-Martínez R. Fernández-Carvajal A. Ferrer-Montiel A. (2022). Paclitaxel *in vitro* reversibly sensitizes the excitability of IB4(−) and IB4(+) sensory neurons from Male and female rats. Br. J. Pharmacol. 179, 3693–3710. 10.1111/bph.15809 35102580 PMC9311666

[B120] Villalba-Riquelme E. de la Torre-Martínez R. Fernández-Carvajal A. Ferrer-Montiel A. (2025). Oxaliplatin reversibly and differentially affects electrogenic activity of small IB4(+) of Male and female rat sensory neurons. Biomed. Pharmacother. 183, 117849. 10.1016/j.biopha.2025.117849 39914991

[B121] Wang H. Siemens J. (2015). TRP ion channels in thermosensation, thermoregulation and metabolism. Temperature 2, 178–187. 10.1080/23328940.2015.1040604 27227022 PMC4843888

[B122] Wang Y. Feng C. He H. He J. Wang J. Li X. (2018). Sensitization of TRPV1 receptors by TNF-α orchestrates the development of vincristine-induced pain. Oncol. Lett. 15, 5013–5019. 10.3892/ol.2018.7986 29552137 PMC5840530

[B123] Wang C. Chen S. Jiang W. (2022). Treatment for chemotherapy-induced peripheral neuropathy: a systematic review of randomized control trials. Front. Pharmacol. 13, 1080888. 10.3389/fphar.2022.1080888 36618919 PMC9822574

[B124] Wiranata J. A. Astari Y. K. Ucche M. Hutajulu S. H. Paramita D. K. Sulistyoningrum D. C. (2024). Predictive factors of chemotherapy-induced peripheral neuropathy in breast cancer: a decision tree model approach. JCO Glob. Oncol. 10, e2400160. 10.1200/go.24.00160 39541561

[B125] Wu Z. Toro G. Xu G. Dang D. Prater C. Yang Q. (2022). Paclitaxel inhibits KCNQ channels in primary sensory neurons to initiate the development of painful peripheral neuropathy. Cells 11, 4067. 10.3390/cells11244067 36552832 PMC9776748

[B126] Xiong C. Chua K. C. Stage T. B. Priotti J. Kim J. Altman-Merino A. (2021). Human induced pluripotent stem cell derived sensory neurons are sensitive to the neurotoxic effects of paclitaxel. Clin. Transl. Sci. 14, 568–581. 10.1111/cts.12912 33340242 PMC7993321

[B127] Yamamoto S. Egashira N. (2021). Pathological mechanisms of bortezomib-induced peripheral neuropathy. Int. J. Mol. Sci. 22, 888. 10.3390/ijms22020888 33477371 PMC7830235

[B128] Yan H. Dan Y. Nana L. Dongqing G. Junming Z. Xin C. (2026). Incidence and influencing factors of chemotherapy-induced peripheral neuropathy in cancer patients: a systematic review and meta-analysis. Front. Neurol. 17, 1672180. 10.3389/fneur.2026.1672180 41815730 PMC12971429

[B129] Yeo M. Zhang Q. Ding L. A. Shen X. Chen Y. Liedtke W. (2022). Spinal cord dorsal horn sensory gate in preclinical models of chemotherapy-induced painful neuropathy and contact dermatitis chronic itch becomes less leaky with Kcc2 gene expression-enhancing treatments. Front. Mol. Neurosci. 15, 911606. 10.3389/fnmol.2022.911606 36504679 PMC9731339

[B130] Zajaczkowską R. Kocot-Kępska M. Leppert W. Wrzosek A. Mika J. Wordliczek J. (2019). Mechanisms of chemotherapy-induced peripheral neuropathy. Int. J. Mol. Sci. 20, 1451. 10.3390/ijms20061451 30909387 PMC6471666

[B131] Zhang H. Dougherty P. M. (2014). Enhanced excitability of primary sensory neurons and altered gene expression of neuronal ion channels in dorsal root ganglion in paclitaxel-induced peripheral neuropathy. Anesthesiology 120, 1463–1475. 10.1097/ALN.0000000000000176 24534904 PMC4031279

[B132] Zhang Z. Roberson D. P. Kotoda M. Boivin B. Bohnslav J. P. González-Cano R. (2022). Automated preclinical detection of mechanical pain hypersensitivity and analgesia. Pain 163, 2326–2336. 10.1097/j.pain.0000000000002680 35543646 PMC9649838

[B133] Zhao M. Isami K. Nakamura S. Shirakawa H. Nakagawa T. Kaneko S. (2012). Acute cold hypersensitivity characteristically induced by oxaliplatin is caused by the enhanced responsiveness of TRPA1 in mice. Mol. Pain 8, 55. 10.1186/1744-8069-8-55 22839205 PMC3495669

[B134] Zink A. Haferkamp U. Wittich A. Beller M. Pless O. Prigione A. (2022). High-content screening of mitochondrial polarization in neural cells derived from human pluripotent stem cells. Star. Protoc. 3 3, 101602. 10.1016/j.xpro.2022.101602 PMC9361325 35959496

[B135] Żok J. Bieńkowski M. Radecka B. Korniluk J. Adamowicz K. Duchnowska R. (2021). Impact of relative dose intensity of oxaliplatin in adjuvant therapy among stage III Colon cancer patients on early recurrence: a retrospective cohort study. BMC Cancer 21, 529. 10.1186/s12885-021-08183-y 33971834 PMC8112028

